# AU040320 deficiency leads to disruption of acrosome biogenesis and infertility in homozygous mutant mice

**DOI:** 10.1038/s41598-018-28666-6

**Published:** 2018-07-10

**Authors:** Luiz G. Guidi, Zoe G. Holloway, Christophe Arnoult, Pierre F. Ray, Anthony P. Monaco, Zoltán Molnár, Antonio Velayos-Baeza

**Affiliations:** 1 Wellcome Centre for Human Genetics, University of Oxford, Oxford, OX3 7BN UK; 2 Department of Physiology, Anatomy, and Genetics, University of Oxford, Oxford, OX1 3QX UK; 3 Genetic Epigenetic and Therapies of Infertility, Institute for Advanced Biosciences, Inserm U1209, CNRS UMR 5309, Université Grenoble Alpes, Grenoble, F-38000 France; 4 UM GI-DPI, CHU Grenoble Alpes, Grenoble, F-38000 France; 5 Office of the President, Ballou Hall, Tufts University, Medford, MA 02155 USA

## Abstract

Study of knockout (KO) mice has helped understand the link between many genes/proteins and human diseases. Identification of infertile KO mice provides valuable tools to characterize the molecular mechanisms underlying gamete formation. The *KIAA0319L* gene has been described to have a putative association with dyslexia; surprisingly, we observed that homozygous KO males for *AU040320*, *KIAA0319L* ortholog, are infertile and present a globozoospermia-like phenotype. Mutant spermatozoa are mostly immotile and display a malformed roundish head with no acrosome. In round spermatids, proacrosomal vesicles accumulate close to the acroplaxome but fail to coalesce into a single acrosomal vesicle. In wild-type mice AU040320 localises to the *trans*-Golgi-Network of germ cells but cannot be detected in mature acrosomes. Our results suggest AU040320 may be necessary for the normal formation of proacrosomal vesicles or the recruitment of cargo proteins required for downstream events leading to acrosomal fusion. Mutations in *KIAA0319L* could lead to human infertility; we screened for *KIAA0319L* mutations in a selected cohort of globozoospermia patients in which no genetic abnormalities have been previously identified, but detected no pathogenic changes in this particular cohort.

## Introduction

Spermatogenesis is a complex developmental process by which mammalian spermatozoa, the male haploid germ cells, are produced from diploid spermatogonia. It includes multiple cellular steps and, for simplification, is divided into specific stages (I–XII in mouse) according to the grouping or association of the different cell types detected after cross-section of the seminiferous tubules^1^. Proliferating spermatogonia line the epithelial wall of the tubules and divide by mitosis to produce spermatocytes which, in turn, undergo meiosis and become round spermatids. These, through a process known as spermiogenesis involving morphologically different and identifiable steps (1–16 in mouse), transform into mature spermatozoa containing a tail (flagellum) and a unique organelle called the acrosome. These germinal cell types are arranged in definite associations or stages (I–XII in mouse), containing one or two spermatids, which constitute the cycle of the seminiferous epithelium^2–4^.

The acrosome is a giant vesicle of secretion bound to the anterior part of the spermatozoon nucleus containing digestive glycosylated enzymes such as hyaluronidase and acrosin that break down the outer protective layers of the ovum, made of the follicular cells and the *zona pellucida*, allowing the sperm to reach the ooplasm. Acrosomes vary in size and morphology across mammals, from the sickle shape in rodents to cap-like structure in humans, although their basic structure is well-conserved^5–7^. The formation of the acrosome occurs during spermiogenesis which can be subdivided in four phases^1,2,6,8^. In the Golgi phase, during early spermatid differentiation (steps 1–3 in mouse), numerous proacrosomal vesicles derived from *trans-*Golgi stacks fuse on one side of the nuclear envelope to form a single large acrosomal granule. As vesicles accumulate during the cap phase (steps 4–7), the acrosome starts to expand along the nuclear surface and tapers into a cap-like compartment. During the acrosome phase (steps 8–12) there is a complex series of morphological changes leading to elongation of the sperm head culminating at the maturation phase (steps 13–16) with the removal of the excess of cytoplasm and formation of the free spermatozoon.

Abnormalities in acrosome development can lead to severe morphogenetic defects in spermatozoa leading to infertility and in humans it typically results in a rare but severe disorder called globozoospermia, estimated to affect less than 0.1% of the infertile male population and with a clear genetic component in multiple cases^9–11^. Globozoospermic spermatozoa are characterised by a rounded head lacking an acrosome and often accompanied by abnormal nuclear condensation and impaired sperm motility^9,10^. In humans, mutations in only two genes have been clearly demonstrated to cause globozoospermia: *DPY19L2*, in which most of known causative mutations have been found^12–17^, and *SPATA16*^18–20^. Putative mutations have also been reported in two other genes: *PICK1*^21^ and *ZPBP1*^22^, but their implication in human globozoospermia is unconvincing^10,11^. In mice, several genes have been associated to defects that parallel human globozoospermia following genetic knockout^10,23,24^. The proteins encoded by these genes have been shown to function in different cellular compartments and affect distinct stages of acrosome biogenesis such as protein processing in the ER (GBA2, HSP90b1), trafficking of vesicles from the Golgi apparatus (PICK1, GOPC, CSNK2a2, SMAP2), normal fusion of acrosomal vesicles (VPS54, HRB), integrity of acrosomal matrix (ZPBP1/2), and interaction of the acrosome with the acroplaxome (SPACA1) or the nuclear envelope (DPY19L2), amongst others.

Here, we report the identification of *AU040320* as a novel gene leading to globozoospermia when disrupted in mice. The human homologous *KIAA0319L* gene has been associated with susceptibility to the neurodevelopmental disorder dyslexia^25^ and to parameters such as language impairment and IQ in autism^26,27^. This gene is widely expressed in the organism, but the function of the encoded protein is largely unknown. We (Velayos-Baeza *et al*., in preparation) and others^28^ have shown that KIAA0319L/AU040320 is a highly-glycosylated type I transmembrane protein that can localise to the plasma membrane, endosomes, Golgi and *trans*-Golgi network (TGN). It follows the classic clathrin trafficking pathway (Velayos-Baeza *et al*., in preparation) and functions as a receptor for the infection of adeno-associated virus into cells^28^.

Our experiments show that absence of AU040320 leads in male germ cells to round-headed spermatozoa that lack an acrosome. This impairment is apparent during the early stages of spermatid differentiation as proacrosomic vesicles fail to fuse with one another and no acrosomal granule is formed. Given the limited understanding of genetic mutations leading to globozoospermia in humans, we have also screened for exonic changes in *KIAA0319L*, in 16 patient samples already screened negative for deletions in the *DPY19L2* gene, but have not found any pathogenic *KIAA0319L* mutation.

## Results

### Genetic deletion of *AU040320* leads to male infertility

**Figure 1:**
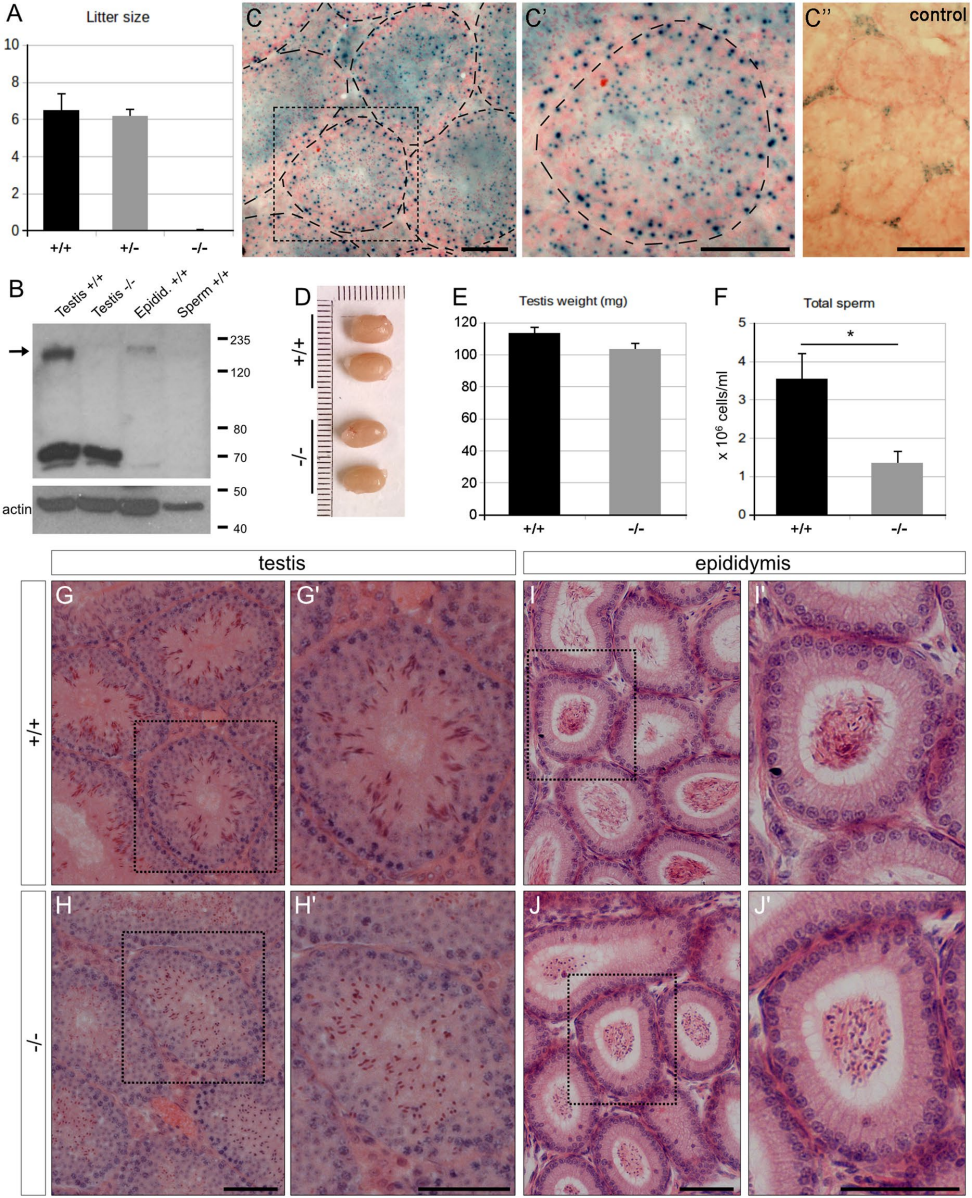
Absence of AU040320 leads to male infertility. (**A**) Average number of pups born from matings with males wild-type (n = 10), heterozygous (+/−; n = 12) and homozygous (−/−; n = 12) for the *AU040320* KO allele. (**B**) Analysis by Western blotting demonstrating expression of AU040320 in testis of wild-type (+/+) and its absence in *AU040320* KO mice (−/−). The protein is also detected in wild-type lysates of the epididymis but not in isolated spermatozoa. Actin was used as loading control. The cropped blots are displayed; full-length blots and Ponceau staining of membranes are shown in Supplementary Fig. S5A. 20 µg loaded per lane. (**C**) X-gal staining of testis sections of *AU040320* −/− mice carrying the *lacZ* reporter gene. Representative image shows the ß-galactosidase activity (blue dots) resulting from the reporter gene which can be seen in the majority of cells of seminiferous tubules (inset, C’). As expected, wild-type control samples do not display any signal in seminiferous tubules, although some likely unspecific signal is detected in the interstitial region corresponding to the Leydig cells (C”). Nuclei are counterstained with Nuclear FastRed. Tubules are outlined in KO samples (C, C’). (**D**) Images of testes of wild-type and *AU040320* −/− mice show no major morphological abnormalities or differences in size. (**E**) Average weight of individual testis is comparable between wild-type and mutant mice (n = 8). (**F**) Quantification of sperm number (x10^6^ cells/ml) revealed a reduction in the number of spermatozoa released from the cauda epididymis of *AU040320* KOs (*denotes p < 0.05; n = 3 per genotype). (**G–J**) Bouins-fixed, H&E-stained histological sections of wild-type and *AU040320* KO mice from testis (**G**,**H**) and epididymis (**I**,**J**), with selected individual tubules shown at larger magnification (G’-J’), including seminiferous tubules containing spermatids in late stages (13–16) of development (G’, H’). Scale bars: 25 μm for all panels.

As part of our investigations into the putative role of the *KIAA0319* and *KIAA0319L* genes in dyslexia susceptibility, we have recently reported the generation, neuro-anatomical analysis and behavioural characterisation of knock-out (KO) mice for the homologous genes *D130043K22Rik*^29^ and *AU040320*^30^, respectively. Interestingly, homozygous *AU040320* KO males, carrying the *tm1b* allele, were infertile despite the presence of vaginal plugs in females, while heterozygous males showed no reproductive deficits (Fig. 1A). The same phenotype was presented by males carrying a different KO allele, the *KO1* or *tm1a* allele, which has been shown to produce some residual detectable levels of protein in brain samples^30^. Mutant females do not display any obvious fertility abnormalities (data not shown). We decided to characterise in more detail the cellular and molecular aspects of this phenotype, using KO males carrying the *tm1b* allele, in order to gain some insight into the function of the AU040320/KIAA0319L protein.

First, we confirmed expression of the AU040320 protein in the mouse reproductive system by Western blot. As previously described for the analysis of brain and other mouse tissue samples^30^, we used a custom antibody raised in guinea pig against the C-terminal cytosolic fragment of human KIAA0319L protein (KL-FCt-C1 or #78) to investigate the presence of the AU040320 protein in lysates derived from testes, epididymal tissue and spermatozoa of wild-type mice, comparing them with lysate derived from *AU040320* homozygous KO mice as a negative control (Fig. 1B and Supplementary Fig. S5). The AU040320 protein, with an apparent size of ~150 kDa, was clearly detected in the wild-type and not in the KO testes samples. A signal at ~70 kDa most probably originated from unspecific cross-reactivity of the antiserum was detected in both samples, as previously reported for other tissues^30^. An alternative interpretation is that this band could represent a different AU040320 isoform not affected by the gene targeting present in the KO mice used for this work. However, the expression data for this gene available in the databases would not support this option since, although there are several transcript variants described, none with a specific tissue expression, all protein-coding variants would be affected in these KO mice. Similar results were obtained after detection with a different specific anti-KIAA0319L antibody (ProteinTech #21016-1-AP, see Materials and Methods) using testis lysates, with the same specific AU040320 band present only in wild-type samples, and some other unspecific bands, different than with #78 antiserum above (Supplementary Fig. S5B). A clearer pattern was detected in epididymal tissue, where only the specific AU040320 band was detected, although the signal appears much weaker than in testes. Finally, no specific signal was detected in lysates from spermatozoa isolated from the cauda epididymis (Fig. 1B); the actin reactivity seemed to be also much lower than in other samples, although the total loaded protein as indicated by Ponceau staining of the membrane appeared uniform in all lanes (Supplementary Fig. S5A).

In order to gain a better picture of the cell types in which *AU040320* is expressed we took advantage of the *lacZ* reporter gene present in the KO allele of *AU040320* mutants^30^, which is expressed under the control of the endogenous *AU040320* promoter, and performed X-gal staining in testis sections. These experiments revealed a widespread distribution of ß-galactosidase positive cells, with its characteristic dotted blue signal coming from most cells in seminiferous tubules (Fig. 1C). These results are in line with available transcriptomic data reporting that *AU040320* transcripts are detected in isolated supporting cells and in germ cells, with its expression peaking at the onset of spermatid differentiation^31–33^.

Examination of the gross anatomy of testes from AU040320-deficient mice revealed they did not display major abnormalities as both size and weight were similar to those of wild-type samples (Fig. 1D,E). Histological analyses of testis sections stained with hematoxylin and eosin also showed that the overall structure of seminiferous tubules and epididymides was largely unaffected (Fig. 1G–J). There was no obvious change in any of these structures observed from these images, except the shape in late stage spermatids (Fig. 1G,H) and in spermatozoa (Fig. 1I–J) (see next section), but a considerable reduction of the number of spermatozoa in *AU040320* −/− mice was identified when the number of cells isolated from the cauda epididymis was quantified and compared to wild-type samples (Fig. 1F). Additionally, the motility of these epididymal spermatozoa after examination by live microscopy was found to be largely absent, although some motile cells were also observed (data not shown).

We analysed potential deficits in cell division and meiosis in germ cells by immunofluorescence. Cells from seminiferous tubules of mutant and wild-type mice were dissociated and immunostained with an antibody against phospho-γ-H2AX to label the XY body and detect potential recombination problems during prophase 1 progression, and the specific stage of meiosis was identified by labelling synaptonemal complex 3 (SCP3). These stainings revealed similar patterns in both wild-type and mutant samples (Supplementary Fig. S1), suggesting meiotic progression of spermatocytes and chromatid recombination over the course of meiosis are not majorly altered in *AU040320* mutants. These results indicate that the reduced epididymal count observed in AU040320-deficient samples is likely not due to major problems in meiosis but may be explained by alterations during spermiogenesis or between the testis and the cauda epididymis.

### Absence of AU040320 leads to abnormal sperm morphology with globozoospermia-like features

From our initial analyses in hematoxylin and eosin (H&E) stained sections above (Fig. 1G–J), we observed that *AU040320*-null spermatozoa appeared to have a general morphology different from that of wild-type ones, displaying a roundish head shape. In order to examine this in more detail, we performed a series of experiments to dissect the overall organisation of these cells and their ultrastructure.

**Figure 2:**
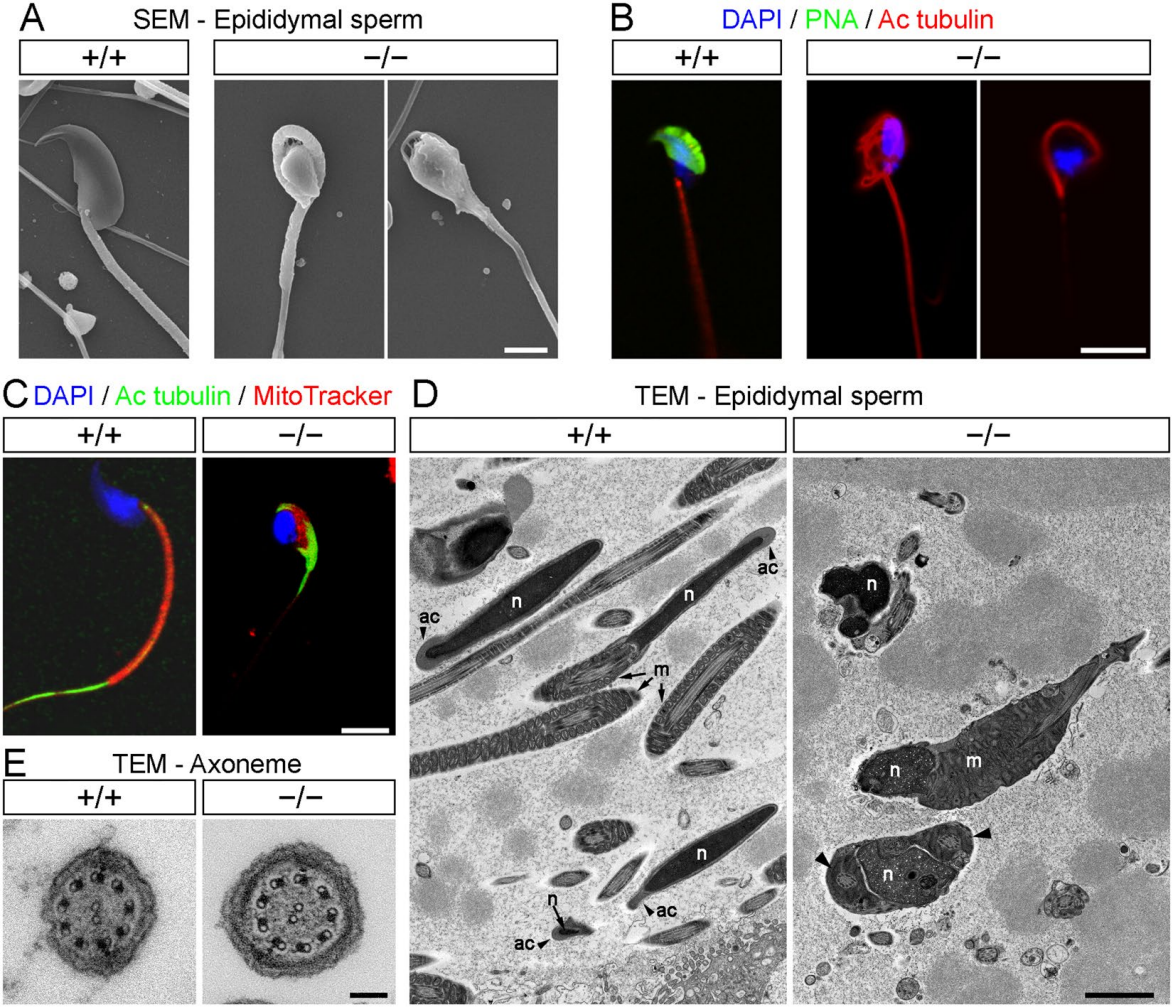
AU040320-deficient spermatozoa exhibit globozoospermia-like features. (**A**) SEM images of wild-type and AU040320-deficient spermatozoa demonstrate the rounded nuclear shape and lack of acrosome in all spermatozoa analysed (100% spermatozoa of each animal; n = 5). (**B**) Immunostaining of dissociated epididymal spermatozoa shows that cells from AU040320-deficient mice have rounded nuclei (DAPI, blue) instead of characteristic sickle shape, lack an acrosome (PNA, green) and tails (acetylated tubulin, red) coiled around the sperm head. (**C**) Mitochondrial midpiece (MitoTracker, red) in mutant spermatozoa is disarranged instead of forming the normal elongated thin structure at the base of the cell nucleus and extending with the flagellum (acetylated tubulin, green). (**D**) Ultrastructure of spermatozoa obtained with TEM reveals the absence of the acrosome in rounded-head mutant spermatozoa in all cases, with disarranged mitochondria and coiled tail in some instances (right panel, arrowheads). Additional examples of images from these analyses are shown in Supplementary Fig. S2. Ac, acrosome; m, mitochondria; n, nucleus. (**E**) Normal ciliary axoneme is detected in spermatozoa from *AU040320* KO mice, with the microtubular organisation containing the 9 outer doublets and the central pair of microtubules. Scale bars: (**A**,**D**) 2 μm; (**B,C**) 5 μm; (**E**) 100 nm.

Scanning electron microscopy (SEM) images revealed that cells produced by *AU040320 −/−* mice did not have the characteristic sickle-shaped head found in mouse sperm, but instead displayed a roundish morphology and a smaller head size (Fig. 2A). All mutant spermatozoa analysed displayed this morphology. The sperm midpiece also appeared disarranged in these images and many tails were found coiled around the head. We then visualised the different subdivisions of spermatozoa with specific markers by immunostaining. Labelling the acrosome with lectin (peanut agglutinin, PNA; green) revealed this structure to be clearly visible in wild-type samples but completely absent in spermatozoa from AU040320-deficient mice (Fig. 2B). Sperm tails were identified with an antibody against acetylated tubulin (Ac tubulin) and found to be present in both conditions, although they appeared coiled in mutants as identified in our SEM images. We also observed some severe midpiece dysplasia in mutant sperm using MitoTracker as these cells did not display the characteristic compact and structured sheath extending from the nucleus observed in wild-type controls (Fig. 2C). Similar DAPI signal from nuclei was detected in sperm from both wild-type and mutant mice (Fig. 2B,C).

Transmission electron microscopy (TEM) confirmed the absence of the acrosome, alongside the rounded cell nucleus and mitochondrial dysplasia (Fig. 2D and Supplementary Fig. S2). Interestingly, the density of the nuclear signal in some of the KO sperm heads seemed to be different than in wild-type samples, which might indicate a variable degree of alteration in nuclear condensation. This topic was not a focus of our original study and was not further investigated. Given the motility impairment observed in mutants, the axoneme of the flagellum was examined but its 9 + 2 microtubular structure appeared intact, with dynein arms clearly visible (Fig. 2E). Altogether, these results show that total loss of AU040320 leads to abnormal head morphology and absence of the acrosome, features that parallel the morphological defects observed in human cases of globozoospermia^9^.

### Impaired acrosome biogenesis in *AU040320* KO mice

The abnormal morphological characteristics described above suggest a morphogenetic defect during spermatid development in *AU040320 −/−* mice. Given globozoospermia typically results from abnormalities in acrosome formation, we used PNA labelling of testes sections to investigate the developmental course of acrosome biogenesis in *AU040320* mutants.

**Figure 3:**
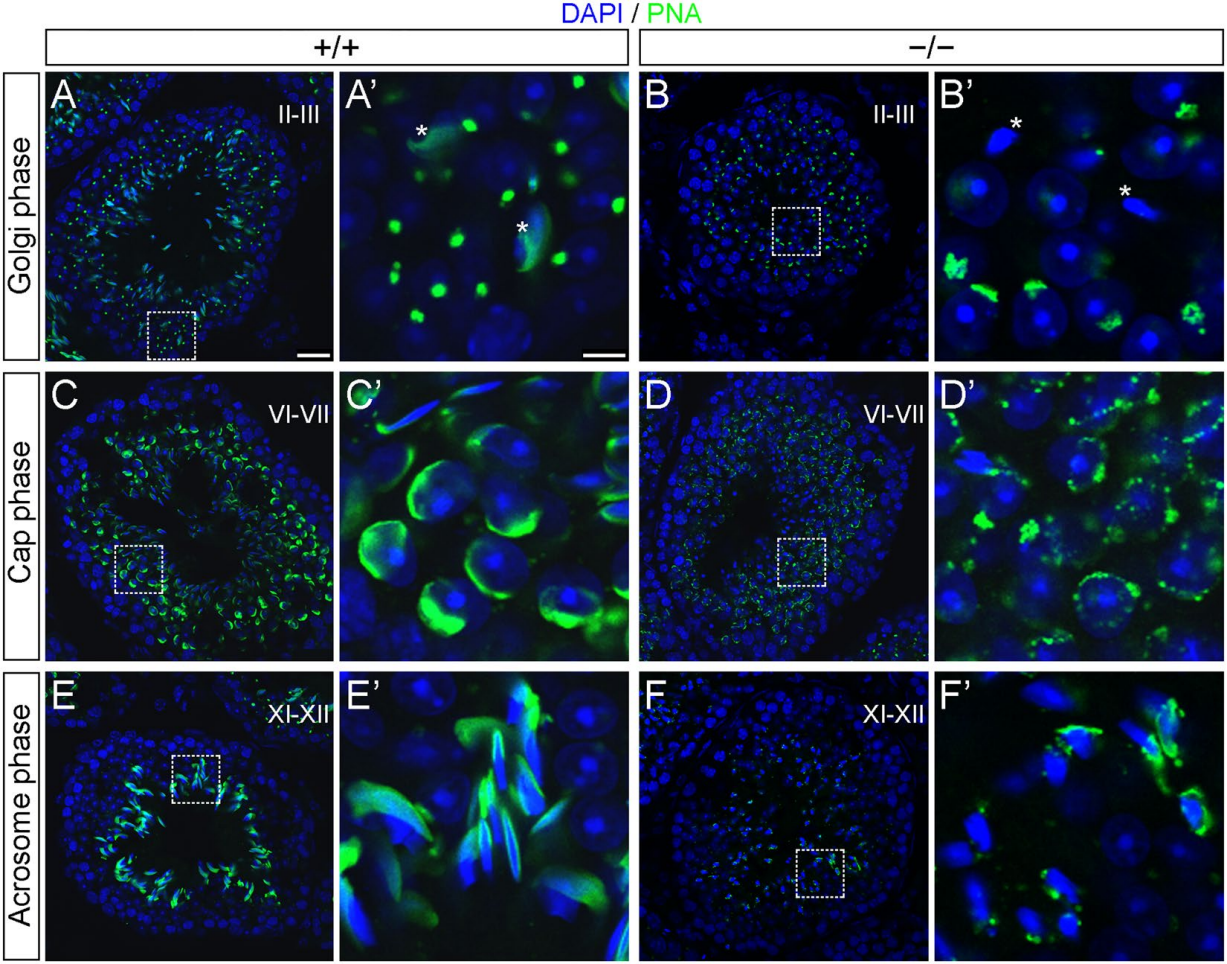
Acrosome formation is impaired in AU040320-deficient mice. Adult testis sections from wild-type (wt, left panels) and KO (null, right panels) samples stained with PNA (green) to label developing acrosome and DAPI for cell nuclei (blue) to identify stage-dependent cell types; development stage of shown tubules appears in the main panels and that of analysed spermatids in inset panels (Golgi/Cap/Acrosome phases) is shown on the left. PNA staining in wild-type spermatids in the Golgi phase accumulates homogeneously as a single structure (**A**), whilst spermatids from AU040320-deficient mice have a diffuse pattern (**B**). In the cap phase, wild-type spermatids display the characteristic thin layer of acrosomal caps (**C**) while mutant spermatids have a punctate PNA staining that does not form a single acrosomal vesicle (**D**). In acrosome phase, PNA-labelled acrosomes elongate along with cell nuclei in wild-type testis samples (**E**) but this pattern is not observed in mutant samples (**F**), where PNA staining appears disarranged and absent in many cases. Similar pattern is also observed in maturation phase; asterisks in inset panels A’ and B’ indicate examples of spermatids in maturation phase present in these tubules. For each condition, n = 5. Scale bars: main panels 25 μm, insets 5 μm.

In wild-type tubules, the developing acrosomes in Golgi phase spermatids displayed the expected pattern clustering on one face of the cell nucleus (Fig. 3A) but PNA staining in mutant spermatids appeared less uniform and more sparse, without forming a single homogeneous compartment (Fig. 3B). These defects were more evident in the cap phase, as acrosomes appeared in a punctated pattern along the nuclear surface instead of the characteristic cap-like layer enveloping the nucleus seen in wild-types (Fig. 3C,D). These defects persisted as spermatids started elongating during the remodelling of the sperm head in acrosome and maturation phases, with acrosomal content accumulating in a disarranged pattern that sometimes appears as a continuous PNA-labelling and with many cells apparently lacking any signal (Fig. 3E,F; see also Fig. 3A’,B’). These results indicate that *AU040320* KO leads to a disruption of the normal formation of the acrosome as early as in the Golgi phase.

### Absence of AU040320 leads to failure in fusion of proacrosomal vesicles

The formation of the acrosome involves the fusion of proacrosomal vesicles typically derived from the *trans*-Golgi for the formation of a single, large acrosomal vesicle attached to the nuclear envelope and the acroplaxome on the Golgi face of the cell nucleus^5,7^. To understand the failure in acrosome formation in more detail, we examined the ultrastructure of developing spermatids using TEM.

**Figure 4:**
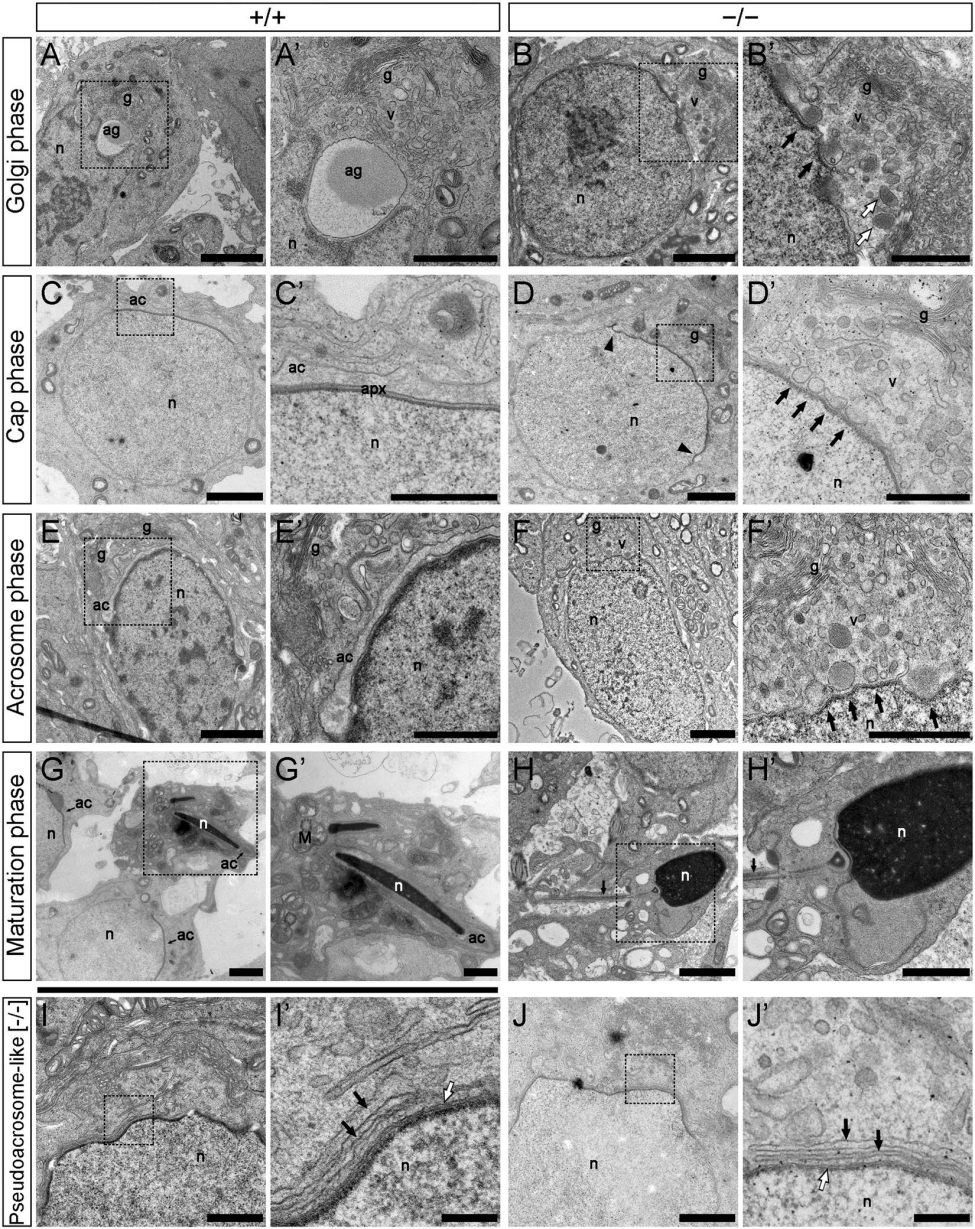
Proacrosomal vesicle fusion is impaired in *AU040320*-null mice. TEM images of developing spermatids in seminiferous tubules of wild-type and mutant mice. Wild-type sperm exhibit the characteristic developmental path of the acrosome, with proacrosomal vesicles fusing together to form a large acrosomal granule (**A**) which becomes flattened along the acroplaxome and the nuclear face in the cap phase (**C**), extends further over the elongating nucleus in the acrosome phase (**E**), and matures into a structure covering nearly all the now heavily condensed nucleus in the maturation phase (**G**). A couple of spermatocytes in cap phase appearing alongside those in maturation phase are shown in panel G. In mutant spermatids, vesicles containing proacrosomal material (B’, white arrows) are observed approaching the nucleus and forming multiple points of indentations on it (B’, black arrows) but fail to coalesce into a single acrosomal granule. As they develop, mutant round spermatids continue to show no acrosomal structure despite the continuous arrival of proacrosomal vesicles (D’, arrows); the curvatures observed at the edge of the acroplaxome in these cells (**D**, arrowheads) may indicate the presence of the manchette. A similar pattern appears in mutant spermatids in the acrosome phase (**F**) which display no developing acrosome, despite the more elongated cell morphology and the apparent presence of the acroplaxome revealed by the flattened nuclear surface (F’, arrows), while larger proacrosomic vesicles can be observed aligning to the acroplaxome. At maturation phase, mutant spermatids show a condensed globular, not elongated, nucleus but no acrosome (**H**), although an apparently normal developing tail (arrow) can be detected. While mutant spermatids do not develop an acrosome, they appear on occasions, at the cap/acrosome phases, to contain a pseudoacrosome-like structure (**I,J**) consisting of saccules organised in a layered fashion (black arrows) in close proximity to the acroplaxome (white arrows). n, nucleus; g, Golgi; ac, acrosome; apx, acroplaxome; ag, acrosomal granule; v, proacrosomal vesicles; M, midpiece of developing sperm tail. For each condition, n = 5. Scale bars: (**A–H**) 2 μm, insets 1 μm; (**I,J**) 1 μm, insets 200 nm.

Wild-type spermatids displayed the characteristic progression of acrosome development with the formation of the acrosomal granule (Fig. 4A) and its subsequent thinning and growth into a cap-like structure (Fig. 4C,E). However, no acrosomal granule was detected in spermatids from *AU040320*-null mice. Instead, during the Golgi phase vesicles accumulated closely associated with the nuclear envelope but without forming a single structure (Fig. 4B). In some cases, some slightly larger vesicles were visible but these never came to form the expected large granule-like compartment (Fig. 4B’, white arrows). In the cap phase, vesicles continued to cluster along the nuclear surface adjacent to the nuclear envelope but did not give rise to a homogeneous compartment as seen in wild-type mice (Fig. 4D). Some mutant spermatids exhibited curvatures on the edges of the acroplaxome suggesting the manchette, a transient microtubular structure that generates a complex interplay of contractile forces in association to the acroplaxome and acrosome^34^, may be present (Fig. 4D, arrowheads). The golgi apparatus (g) appeared unaltered in KO mice (Fig. 4D). In later stages of development, as mutant spermatids attempted to elongate, the same pattern of accumulation of small vesicles on the nuclear surface was observed (Fig. 4F). In addition, the flattening of the nuclear face observed in some cases indicates the acroplaxome appears largely unaffected (Fig. 4F). Morphology at maturation phase, similarly as shown for the mature sperm (Fig. 2D and Supplementary Fig. S2), was clearly different between spermatids from wild-type (Fig. 4G) and KO (Fig. 4H) mice, with no acrosome and globular nucleus in mutant spermatids; nuclear condensation and tail formation are detected.

Given acrosome formation and sperm head morphogenesis involve a highly complex developmental program, we investigated whether these deficits in vesicle fusion were accompanied by abnormalities in other structures important for acrosome formation. Thus, we examined the basic morphology of Sertoli cells, with which developing spermatids interact via adhesion molecules and actin-based scaffolds^35,36^, by immunolabelling with a specific antibody (β-tubulin III). We found no major abnormalities in the cytoskeletal structure of these cells as they were arranged uniformly and extended into the luminal region similarly to wild-type samples (Supplementary Fig. S3A). In addition, we decided to assess the integrity of the sperm manchette by immunostaining with β-tubulin and confirmed that manchettes in mutant cells were largely comparable to those of wild-type samples (Supplementary Fig. S3B). These results indicate that absence of AU040320 does not lead to major cytoskeletal abnormalities in supporting structures such as Sertoli cells but the possibility of more subtle defects cannot be ruled out.

Although no acrosome was detected in spermatids from *AU040320* mutant mice in all samples examined, a pseudoacrosome-like structure in a multi-layered membrane arrangement lining the nuclear envelope was observed in a number of cells in cap and acrosomal phases (Fig. 4I,J), although this point was not investigated in more detail. Such features have been observed in other globozoospermia-like mouse mutants and may result from the displacement of the ER and its attachment to the acroplaxome^37,38^. These structures, as suggested above, are most probably not associated with the apparently continuous PNA-signal detected in some acrosome-phase spermatids from mutant mice (Fig. 3F). These observations suggest that impaired acrosome formation in spermatids from *AU040320 −/−* mice may result from a failure in the normal fusion or transport of proacrosomal vesicles.

### AU040320 localises to the trans-Golgi network

The KIAA0319L protein, the human homolog of AU040320, has been previously reported to localise or be closely associated to the plasma membrane, the *cis*-medial Golgi, the TGN and endosomes in HeLa cells^28,39^. In order to verify the specific distribution of this protein in germ cells of wild-type mice, immunohistochemistry was performed in testis sections using two different antibodies recognising distinct regions of the AU040320 protein and already used in Western blot analysis (Supplementary Fig. S5) (see Materials and Methods and Table 1). KO samples served as negative controls for antibody specificity.

**Table 1:** Antibodies and other detection reagents used in immunostaining experiments.

Antibody/reagent^a^	Conjugate^b^	Species^c^	Dilution	Company	Cat. No.
anti-KIAA0319L (#78)		Guinea pig	1:1000	(custom made)	KL-FCt-G1
anti-KIAA0319L (Rb-AU)		Rabbit	1:1000	ProteinTech	21016-1-AP
anti-acetylated Tubulin [6-11B-1]		Mouse; M	1:1000	Sigma	T7451
anti-beta-Tubulin [TUB2.1]	CY3	Mouse; M	1:200	Sigma	C4585
anti-beta-III Tubulin [TUJ1]		Mouse; M	1:1000	Abcam	ab14545
anti-phospho-Histone H2A.X (Ser139) [JBW301]		Mouse; M	1:500	Millipore	05–636
anti-SCP3		Rabbit	1:500	Abcam	ab15093
anti-rat TGN38		Sheep	1:200	BioRad	AHP499
anti-Sheep IgG (H + L)	Biotin	Rabbit	1:100	Vector Laboratories	BA-6000
anti-Guinea pig IgG (H + L)	AF-488	Goat	1:1000	Molecular Probes	A11073
anti-Guinea pig IgG (H + L)	AF-568	Goat	1:1000	Molecular Probes	A11075
anti-Rabbit IgG (H + L)	AF-488	Goat	1:1000	Molecular Probes	A11008
anti-Rabbit IgG (H + L)	AF-568	Goat	1:1000	Molecular Probes	A11011
anti-Mouse IgG (H + L)	AF-488	Goat	1:1000	Molecular Probes	A11001
anti-Mouse IgG (H + L)	AF-568	Goat	1:1000	Molecular Probes	A11004
Streptavidin	AF-488		1:500	Molecular Probes	S11223
Lectin PNA	AF-488		1:1000	Molecular Probes	L21409
Lectin PNA	AF-568		1:1000	Molecular Probes	L32458
MitoTracker	Deep Red FM		1:1000	Molecular Probes	M22426

^a^Anti-KIAA0319L antibodies recognise mouse AU040320 protein (referred to as anti-AU040320 in text).

^b^AF, AlexaFluor.

^c^M, monoclonal antibody; all other antibodies are polyclonal.

**Figure 5:**
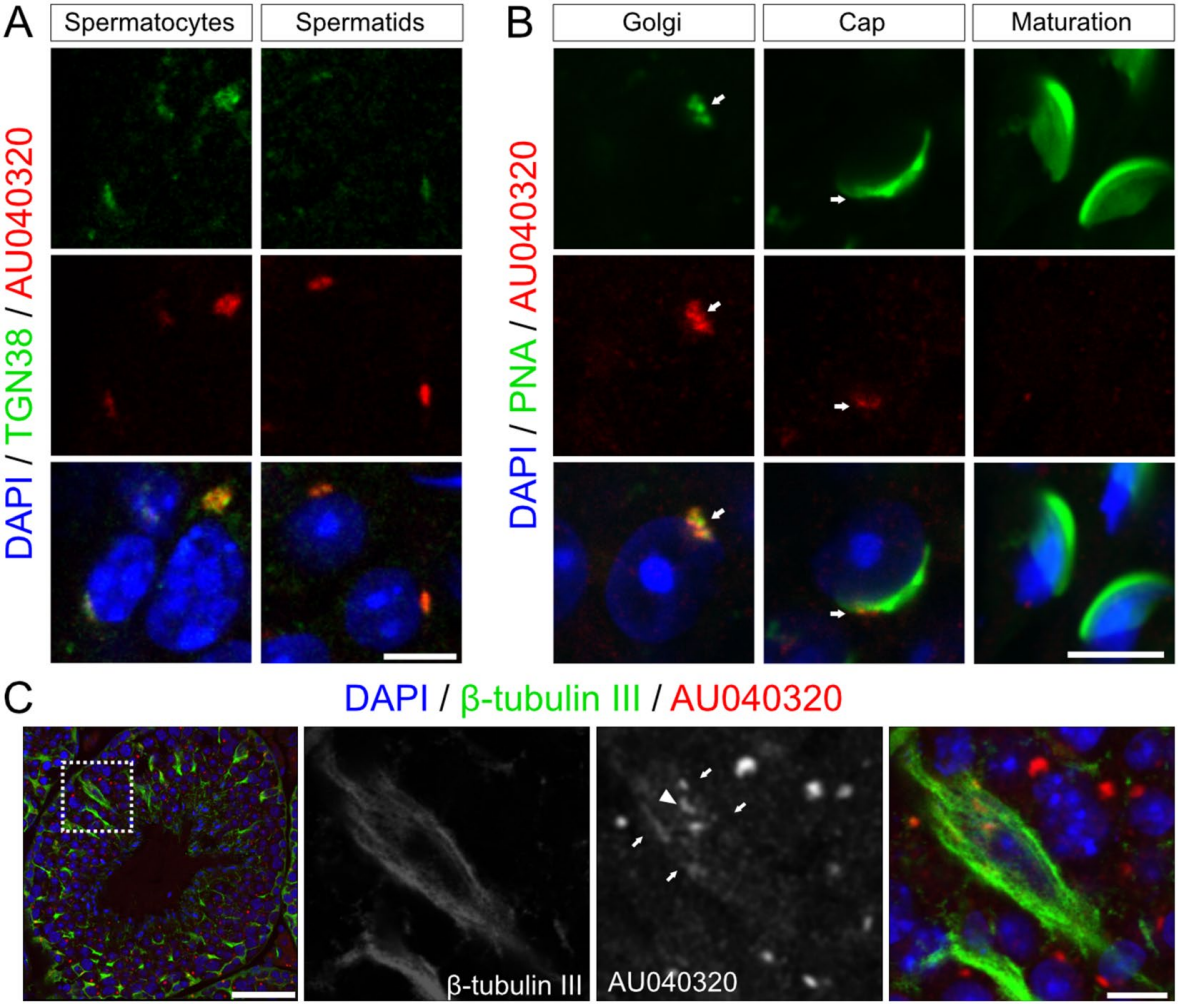
AU040320 localises to the TGN in developing spermatids. (**A**) Double immunohistochemistry for detection of endogenous AU040320 (custom antibody #78, red) and trans-Golgi marker TGN38 (green) shows co-localisation in spermatocytes and round spermatids. (**B**) Comparison of AU040320 localisation (#78, red) with the PNA pattern (green) over the course of acrosome development reveals association of the AU040320 protein with early acrosomal structures in Golgi phase spermatids but this seems to be reduced in Cap phase spermatids (white arrows); no clear signal for AU040320 can be detected at later stages (acrosome-maturation phases). (**C**) AU040320 protein (#78, red) exhibits a pattern with some overlap (arrows) with the structure of Sertoli cells (β-tubulin III, green), including a stronger juxta-nuclear signal compatible with TGN localisation (arrowhead), suggesting AU040320 is also present in these cells. For each condition, n = 3. Scale bars: 5 μm in all panels with the exception of in C (25 μm).

These experiments revealed that both antibodies displayed the same pattern of distribution in spermatocytes and spermatids suggesting the reliable detection of the full-length protein in these cells (Supplementary Fig. S4A–C). Almost no distinguishable signal was detected in the *AU040320-*null samples (Supplementary Fig. S4D–F) suggesting that these antibodies were able to recognise the specific AU040320 signal in the germ cells. Further analysis with antibody #78 showed that most reactivity detected in wild-type seminiferous tubules was lost in the KO samples (Supplementary Fig. S4G–J), except in the interstitial space where Leydig cells still present detectable signal (Supplementary Fig. S4H”,J”); these results might correspond to the non-specific bands detected in testis lysates by Western blot analysis (Fig. 1B and Supplementary Fig. S5). We then used the custom antibody (red) in co-staining experiments with TGN marker TGN38 (green) and found strong co-localisation in both spermatocytes (Fig. 5A, left panels) and round spermatids (Fig. 5A, right panels). AU040320 also seems to associate with early acrosomal structures based on the partial overlap with PNA in spermatids at the Golgi phase (Fig. 5B, left panels). At later stages, after the spreading and thinning of the acrosomal cap, AU040320 and acrosomal proteins appear to occupy different cell compartments as their distribution does not coincide and the AU040320 signal seems to be much reduced (Fig. 5B, middle and right panels). AU040320 was also detected in Sertoli cells, although with a more diffuse pattern in general that could be consistent with a membrane distribution (Fig. 5C; arrows), appearing more intense in the juxta-nuclear region probably representing the TGN (arrowheads). We did not detect the presence of the AU040320 protein in proacrosomal vesicles, although this remains a possibility.

### Mutation screening in human globozoospermia

The identification of the globozoospermia phenotype in mice after null mutations in the *AU040320* gene suggested that mutations in *KIAA0319L* could also lead to globozoospermia in humans. The majority of mutations leading to human globozoospermia identified so far are linked to *DPY19L2*, but several patients remain without a genetic diagnosis^10^. Other genes such as *SPATA16*^18–20^ and *PICK1*^21^ have also been linked to the etiology of globozoospermia. In an attempt to investigate this possibility, we examined genomic DNA from 16 males with globozoospermia for which no genetic abnormalities have been previously identified (see Materials and Methods). Screening of mutations was performed by individual PCR-amplification of each of the 20 coding exons (2 to 21) from *KIAA0319L* from each patient followed by Sanger sequencing. Fragments were amplified to span the entire exons with small flanking intronic regions. This approach could reliably detect point mutations and small insertion/deletions in coding regions and homozygous exonic deletions, but not heterozygous exonic deletions, intronic deletions affecting expression or splicing, or other large rearrangements. Given the infertility phenotype we detected in mice only appears when both copies of the gene are disrupted, we anticipated that, should this gene be involved in human male infertility, it would follow a similar mechanism, that is, affected individuals would present loss-of-function mutations in both *KIAA0319L* alleles. A number of synonymous changes or SNPs were found in these samples but no potentially pathogenic mutations, leading to loss-of-function of the protein, were detected.

## Discussion

The study reported here shows that absence of AU040320 protein in mice leads to male infertility due to gross cell morphology abnormalities characterised by mutant spermatozoa displaying a rounded head and no acrosome, features reminiscent of human globozoospermia^9,10^. Furthermore, these spermatozoa show a coiled tail, with evident mitochondrial disorganisation and impaired motility, features that are common in other globozoospermia models^24^ and likely result from the absence of the acrosome. The formation of the acrosome in AU040320-deficient male mice was disrupted as proacrosomal vesicles failed to fuse and coalesce into a single acrosomal vesicle, leading to an accumulation of vesicles around the nuclear face which, after PNA staining, can occasionally produce an apparently continuous labelling (Fig. 3F’). The abnormalities in head morphology observed in AU040320-deficient spermatozoa, including its smaller size, are likely to be a secondary effect given the importance of the acrosome in head elongation^34^. The alteration in nuclear condensation sometimes reported in globozoospermia was not evaluated in detail in our study, although some of the TEM images of sperm from the KO mice might suggest some differences with the wild-type samples are present. The AU040320 protein localises to the TGN in germ cells but cannot be detected in mature acrosomes suggesting it may be required for the normal formation of proacrosomal vesicles. In addition, we screened the homologous human *KIAA0319L* gene for putative pathogenic changes in a cohort of male patients with globozoospermia by sequencing individual exons but no mutations were identified in this particular cohort. Overall, our study identifies a new player in mouse spermatozoa development with an apparent function in the formation and fusion of vesicles during acrosome formation.

Round-headed sperm heads and absence of the acrosome are the key features of globozoospermia and result from the complex morphogenetic program that takes place during spermiation. During this process, the acrosome appears after the accumulation and fusion of proacrosomal vesicles derived from intracellular trafficking pathways to form a large vesicular organelle that becomes anchored to the nuclear envelope^5^. Loss-of-function mutations in several mouse genes have been found to cause phenotypes resembling human globozoospermia such as *Hrb*^40^, *Gopc*^41^, *Pick1*^42^, *Hsp90b1*^43^, *Dpy19l2*^44^, *Atg7*^45^, *Zpbp1/Zpbp2*^46^, *Spaca1*^47^, *Smap2*^48^, *Mfsd14a*^49^, *Tmf1*^50^ and *GM130*^51^. More rarely, a similar phenotype results from particular missense mutations, such as in the wobbler mouse, homozygous for a mutation (L967Q) in the *Vps54* gene^52^. These genetic models have shown that disruption in a number of different processes during acrosome biogenesis can lead to mouse globozoospermia, from protein processing in the endoplasmic reticulum and fusion of proacrosomal vesicles to the adhesion of acrosome with nuclear structures^10,24^.

The main mechanism underlying the failure of acrosome formation in AU040320-deficient spermatids appears to be a failure in the fusion of proacrosomal vesicles after they are formed in the Golgi. Amongst the other globozoospermia mouse mutants, the abnormalities observed in *AU040320*-null mice at this particular step resemble, in different degrees, those observed for KO mice for *Hrb*^40^, *Gopc*^41^, *Mfsd14a*^49^, *Tmf1*^50^, *GM130*^51^ and *Pick1*^42^, or in the *Vps54-L967Q* mouse^52^. In these cases, including *AU040320*, proacrosomic vesicles fail to coalesce into a single acrosomal structure and, instead, accumulate along the nuclear surface without clustering. However, the specific points in which this disruption occurs may be different in some or all of these models as several of them do present enlarged vesicles or even anomalous acrosomal granules, as it happens in *Pick1* mutants^42^ or in the wobbler mouse^53^. The similarities with some of these globozoospermia mouse mutants can often be equally detected at the level of sperm morphology in general (Fig. 2 and Supplementary Fig. S2) as reported for *Gopc* KO mice^54,55^. Amongst these proteins, HRB has been directly linked to vesicle fusion^40^, GM130 is a *cis*-Golgi protein^56^, TMF1 is a golgin involved in Golgi morphology^57^, VPS54 is a component of the Golgi Associated Retrograde Protein (GARP) complex associated with tethering of endosome vesicles to the TGN^58^, GOPC and PICK1 have been shown to be involved in the budding and trafficking of vesicles from the TGN^42,59,60^, and MFSD14A may be important for protein or lipid glycosylation due to its homology to sugar transporters^49^. This suggests that the failure in the fusion of vesicles in these mutants takes place before the point in which proacrosomic vesicles are expected to fuse. It appears that the impairment in vesicle fusion in AU040320-deficient mice would result from similarly indirect mechanisms.

Failure in acrosome formation can also be a secondary effect, as it seems to occur after invalidation of SPINK2, a serine protease inhibitor which specifically targets acrosin, the main acrosomal protease. Fusion of proacrosomal vesicles is disrupted and leads not to globozoospermia but to azoospermia^61^. In round spermatids SPINK2 was shown to be necessary to prevent acrosine autoactivation during its transit through the TGN and, in its absence, the Golgi apparatus was shown to be destructured, acrosomal vesicles could not fuse and the spermatids arrested their differentiation and were shedded in the tubule often forming symblasts^61^. This suggests that unneutralized acrosine degrades proteins necessary for proacrosomal vesicle fusion, likely including some of the globozoospermia-related proteins mentioned above.

AU040320/KIAA0319L is a transmembrane glycosylated protein with five immunoglobulin-like (Ig-like) domains of the PKD family that lie in the protein’s ectodomain. PKD domains have been linked to cell adhesion^62^ and, during spermatogenesis, important cellular interactions occur between Sertoli cells and developing spermatids via the ectoplasmic specialisation, an actin-based specialised adherens junction that forms between these cell types^63^. This interaction is mediated by transmembrane proteins with adhesive properties, including members of the Ig superfamily as nectins^64,65^. Given the features of the AU040320 protein, a role in these particular junctions would have been a plausible option. However, the abnormalities detected in AU040320-deficient mice differ considerably from those reported in mice with mutations directly affecting the ectoplasmic specialisation. The alterations in this type of mutants do not normally affect round spermatid development around the Golgi phase and tend to emerge from the onset of elongation but, importantly, acrosome problems are not usually described^24^. Although it is possible that AU040320 protein could play some role in the interaction between Sertoli cells and developing spermatids, our results indicate that it would not be essential or as important as that of other proteins described in the ectoplasmic specialisation junction. Therefore, the function of AU040320 relevant for spermatogenesis is most probably required for a different event or step in this process.

The fact that AU040320 appears to localise mainly in the Golgi/TGN and could not be detected in the mature acrosome itself would be consistent with the hypothesis that this protein does not directly operate during vesicle fusion, although it would also be possible that the protein is removed from the acrosome and recycled as suggested for PICK1^42^ or that the protein conformation and/or IF threshold of detection permits its visualization in the Golgi/TGN but not in the acrosome. Thus, the failure of proacrosomal vesicles to fuse in AU040320-deficient spermatids is likely to result from some defect that precedes the fusion events, such as in the processing and formation of vesicles. The delivery and fusion of proacrosomal vesicles in developing spermatids have been shown to be dependent upon the complex molecular machinery required for intracellular trafficking^40,48,66–68^ which includes coating, adaptor, motor, cytoskeletal, Rab or SNARE proteins^69^. Among the proteins associated with abnormalities of sperm acrosome morphogenesis in mouse models with globozoospermia, a number of them have been reported to function in vesicle fusion and/or trafficking^10^. As a membrane protein that can localise to different compartments, AU040320 undergoes intracellular trafficking and we (in preparation) and others^28,39^ have shown it interacts with some key players in this process such as the adaptor proteins AP-1 and AP-2. However, there are no data available that would suggest its connection with this pathway is other than as a cargo protein. Nevertheless, it could be possible that this protein is somehow necessary for the proper formation of proacrosomal vesicles or in the recruitment of cargo proteins that would in turn be required in downstream events leading to acrosomal fusion.

AU040320/KIAA0319L is a type I transmembrane protein with features largely similar to those reported for the KIAA0319 protein^70–72^, with a large extra-cellular/luminal domain and a small C-terminal cytosolic tail (in preparation), a conformation resembling that of transmembrane receptors. While no receptor role has been reported for these proteins, the proteolytic processing described for KIAA0319 has suggested it may have signalling functions^71^. This could also be true for AU040320, and an indirect role through signalling may be behind the altered molecular mechanisms leading to no acrosome formation in the KO mice.

After the identification of *AU040320* as a new gene required for acrosome formation in mice, we explored the possibility that mutations affecting the homologous *KIAA0319L* gene could also cause globozoospermia in humans. However, no pathogenic mutations were identified in the 16 cases investigated, all of which had been previously screened negative for deletions in the *DPY19L2* gene. Whilst no small changes or homozygous deletions were identified in protein-coding regions, it is possible that other genetic abnormalities may be present in the *KIAA0319L* alleles of these patients such as heterozygous duplications or deletions (i.e. copy number variations, CNVs). In contrast to the over ten genes linked to globozoosermia in mice, only two genes have been clearly shown to lead to the disorder in humans: *DPY19L2* which is found mutated in 70–80% of globozoospermic patients and *SPATA16* which was found mutated in only a handful of patients. *PICK1* and *ZPBP1* are also other possible candidates but with very poor genetic evidence^10,11^. It is possible that mutations in these genes are extremely rare and have not yet been identified in human cases of globozoospermia. Alternatively, or additionally, many of these genes would have roles in other cells and tissues that could be essential in human development; anomalies in these genes could therefore induce defects that could range from embryonic lethality to physical or behavioural problems not compatible with active sex life and a will to reproduce. A third explanation may be the presence of slight species differences in the morphogenetic program involving the formation of the acrosome. Also, as human spermatogenesis is not as robust and efficient as mouse spermatogenesis, the human phenotype caused by these gene defects might be more severe such as oligo-astheno-teratozoospermia or azoospermia. Which of these possible explanations, if any, is correct will only be solved if and when similar mutations are found in the homologous human genes.

Despite the widespread expression of AU040320, results available so far indicate that its disruption leads to a marked phenotype mainly in the testis. The Golgi/TGN localisation of this protein and its putative function in vesicle formation and fusion reported here could play a similarly important role in other cells such as neurons. Analysis of the *AU040320*-KO phenotype and of the molecular and cellular mechanisms involved may thus represent a valuable tool to gather insight on the function of this protein in other tissues and organs.

## Materials and Methods

### Experimental animals

Animals were housed with *ad libitum* access to food and water under a 12 h light/dark cycle with temperature and humidity kept constant. Animal generation, maintenance and procedures took place at the Biomedical Services buildings of the University of Oxford. The KO mutant mice used for this work were C57BL/6J-*AU040320*^*tm1b(EUCOMM)Wtsi*^, carrying the *del* allele that conserves the trapping elements of the initial targeting cassette containing the *lacZ* reporter gene and where exon 3 of *AU040320* is also deleted^30^. All animal experiments were authorized and approved by the University of Oxford Animal Care and Ethical Review Committee and UK Home Office (project licence 30/2919) and performed in accordance with the Animals (Scientific Procedures) Act 1986.

### PCR, genotyping and Western blotting

Extraction of genomic DNA and amplification of genomic fragments for genotyping, and preparation of protein lysates and analysis by Western blotting were performed as described previously^29,30^. Tissue samples were collected after culling animals by cervical dislocation and used for preparation of protein lysates or kept at 80 °C until processed. Tissue (testis and/or epididymis) was disrupted and homogenised with a dounce homogeniser using cold RIPA buffer without detergents (50 mM Tris-HCl pH = 7.5, 150 mM NaCl) containing 4X Complete EDTA-free protease inhibitor cocktail (Roche) before being transferred onto a cold Eppendorf tube. The homogeniser was then washed with the same volume of RIPA buffer with 2X detergents (2% NP-40, 1% Na-deoxycholate, 0.2% SDS) which was then mixed with the initial homogenate and left on ice for at least 3 h. Sperm collected from three cauda epididymides (see below) was suspended in 0.1 ml RIPA buffer with protease inhibitor cocktail and left on ice for 1–2 h. Lysates were then spun by centrifugation at 6,000 × *g* for 10 min at 4 °C and supernatant collected into fresh tubes. Lysate protein concentration was quantified using the Pierce BSA Assay (ThermoFisher Scientific). Proteins (20 µg) were prepared with NuPAGE LDS sample buffer with reducing agent and subjected to electrophoresis using NuPAGE Novex 4–12% Bis-Tris gels (Invitrogen) following the manufacturer’s instructions. Gels were transferred onto PVDF membranes (Invitrogen), checked by Ponceau-staining and processed for Western blotting using standard conditions. A custom guinea pig polyclonal antibody against the cytosolic domain (residues 954–1049) of human KIAA0319L protein (KL-FCt-G1 (or #78); Velayos-Baeza *et al*., in preparation) was used for detection of the mouse AU040320 protein (89 identities (93%), 92 positives (95%) over the equivalent 96-residue C-terminal regions). We also used a rabbit polyclonal commercial antibody (Proteintech #21016-1-AP) against the central region, N-terminal to the transmembrane domain, of human KIAA0319L protein (positions 583–932) (327 identities (93%), 347 positives (99%) over the equivalent 350-residue region of mouse AU040320 protein). Mouse monoclonal anti-Actin (BD Transduction Laboratories, #612656) was used as a control. Anti-guinea pig-IgG(H + L) (Zymed, #61–4620) and anti-mouse-IgG(H + L) (BioRad, #170–6516) HRP-conjugated secondary antibodies were used for detection. Immuno-reactive bands were visualised with ECL Plus Western Blot Detection Reagent (GE Healthcare).

### X-gal staining

For detection of ß-galactosidase activity produced by expression of the *lacZ* reporter gene in *AU040320* mutant mice, fresh-frozen samples were cut at 30 μm on a cryostat (Jung CM3000; Leica). Sections were post-fixed for 10 min on ice with 0.2% glutaraldehyde (Polysciences) in PBS and slides washed for 10 min in PBS followed by a detergent rinse (0.02% Igepal, 0.01% Sodium Deoxycholate in PBS) and then incubated overnight at 37 °C in the dark in the detergent solution containing 1 mg/ml X-gal (Thermo Fisher) plus 5 mM potassium ferricyanide and 5 mM potassium ferrocyanide. Next, slides were post-fixed in 4% PFA for 10 min, washed in PBS and distilled water before Nuclear Fast Red counterstaining (Sigma-Aldrich). Images were taken on a light microscope (Leica DMR/DC-500) processed using GIMP Image Editor (GIMP Development Team) and ImageJ (Image Analysis in Java, NIH).

### Hematoxylin and eosin staining

Male mice at least 8 weeks old were culled by cervical dislocation. Testes and epididymides were dissected and fixed by immersion in Bouins fixative (Polysciences) for 6 h before being transferred to 70% ethanol. Samples were washed until excess fixative was removed before being embedded in paraffin. Sections were then cut on a microtome at 6–8 μm and stained for hematoxylin and eosin (H&E). Briefly, this consisted of dewaxing sections in histoclear and rehydrating them in a series of graded steps of ethanol and water. Slides were then immersed in hematoxylin solution and counterstained with eosin before being dehydrated in ethanol, cleared in histoclear and mounted with glass coverslips. Images were taken using a Nikon wide-field TE2000U Microscope.

### Sperm isolation and count

To isolate and quantify the number of mature sperm in the epididymis of adult mice, the cauda epididymis was dissected and placed in 1 ml of warm Dulbecco’s modified eagle medium (Sigma) on a small tissue-culture dish; sperm was released from the tissue by squeezing and puncturing and incubation for 15–30 min at 37 °C. The sperm medium was then diluted and cell density quantified using a hemocytometer. For further processing, sperm medium was harvested and spun at 800 × *g* for 3 min, pellet washed in PBS, spun again and suspended in PBS.

### Meiotic profile analysis of spermatocytes

Analysis of spermatocyte meiotic profile was performed by dissecting seminiferous tubules from adult testes into Minimum Essential Medium (MEM, Sigma-Aldrich) with protease inhibitor cocktail (Roche) and pipetting to release cells. Samples were left on ice and the supernatant collected and spun by centrifugation at 5800 × *g* for 5 min. Cells were suspended with 0.1 M sucrose in water, spread onto slides coated with 1% PFA and placed in a humidity chamber for 2–3 h. Slides were then rinsed with 0.4% Photo-Flo 200 (Kodak), allowed to air dry and used for immunostaining. Non-specific binding sites were blocked by incubating the cells with 0.2% BSA, 0.2% gelatin, 0.05% Tween-20 in PBS for 1 h at room temperature. Cells were incubated with the primary antibodies (Table 1) overnight at 4 °C and secondary antibodies for 2 h at room temperature before mounting.

### Immunohistochemistry and immunofluorescence

For analysis of testis structures and sperm development by immunohistochemistry, adult male mice, at least 8 weeks old, were anaesthetised with pentobarbitone (150 mg/kg, Pentoject, Animal Care Ltd, UK) and transcardially perfused with PBS followed by PFA. Testes were then dissected, stored in PFA overnight, cryopreserved in 30% sucrose in PBS for at least 24 h before being embedded and frozen in OCT compound (TissueTek), and sectioned at 12–18 μm on a cryostat (Jung CM3000; Leica).

For detection of AU040320 protein, sections were processed for antigen retrieval with boiling 10 mM sodium citrate buffer for 1 min followed by 4 min in the same buffer at room temperature. To prevent unspecific binding, sections were incubated in blocking buffer (Chemi Blocker, Millipore, with 0.1% Triton X-100) before incubation with primary antibodies (Table 1) overnight at 4 °C. After PBS washes, slides were incubated with fluorescently-labelled antibodies for 2 h at room temperature and counterstained with DAPI. All antibodies were diluted in blocking buffer and slides were mounted with ProLong Gold Medium (Invitrogen). Images were taken using a Leica TCS SP8-X SMD or a Carl Zeiss LSM710 Confocal Microscope and processed using GIMP Image Editor (GIMP Development Team) and ImageJ (Image Analysis in Java, NIH). The same steps were conducted to inspect the morphology of mature epididymal sperm, isolated as described above, suspended with 0.1 M sucrose in water and spread onto slides. These were then fixed in 4% PFA for 30 min before the immunostaining procedure.

Antibodies used in these applications and their relevant details are listed in Table 1. Antibodies against different forms of tubulin were utilised to detect different structures or cells as typically done in similar studies: anti-acetylated α-tubulin for sperm tails as acetylation of α-tubulin is enriched in flagellar and ciliary axonemes^73^, anti-β-tubulin III for Sertoli cells as this form of tubulin is specific of these cells and neurons^74^, and a generic anti-β-tubulin for the manchette, the skirt-like microtubular structure present in elongating spermatids^75^.

### Scanning electron microscopy

Scanning electron microscopy was carried out based on a previously published protocol^76^. Mature sperm cells were isolated as described above and spread onto coverslips before fixation in 2.5% glutaraldehyde (Polysciences) and 2% PFA (Sigma) in 0.2 M sodium cacodylate buffer (pH = 7.4) at room temperature for 1 h. A secondary fixation was performed with 1% osmium tetroxide in PBS at 4 °C for 1 h before dehydratation in a series of graded ethanol incubations. Drying was performed using hexamethyldisilazane and sputter coated with gold. Images were taken using a JEOL JSM-6390 Scanning Electron Microscope at 5 kV.

### Transmission electron microscopy

To visualise the ultrastructure of mature and developing sperm, transmission electron microscopy was utilised^77^. Epididymis and testis from adult mice were dissected as previously described^78^, cut into 1–2 mm^3^ cubes and fixed for at least 24 h at 4 °C in 2.5% glutaraldehyde (Polysciences) and 2% PFA (Sigma) in 0.1 M 1,4 Piperazine bis(2-ethanosulfonic acid) (PIPES) buffer (pH = 7.2, Sigma-Aldrich). Samples were washed in 0.1 M PIPES buffer with 50 mM glycine before a second fixation step with 1% osmium tetroxide in 0.1 M PIPES for 1 h at 4 °C and a tertiary fixation in 2% uranyl acetate for 2 h at 4 °C. Tissue was then dehydrated in gradient steps of ethanol and embedded in epoxy-resin. Ultrathin sections were cut in an ultramicrotome (Ultracut 7, Leica) and imaged using a Tecnai 12 (FEI) Transmission Electron Microscope operated at 20 kV with a OneView CMOS camera (Gatan). Images were adjusted for brightness/contrast with GIMP Image Editor (GIMP Development Team).

### Mutation screening in globozoospermia patients

Sixteen males with primary infertility due to globozoospermia were recruited in France and Tunisia, and screened for mutations in the *KIAA0319L* gene. The spermatozoa in these patients were characterised in the Ray/Arnoult laboratory as previously described^14^. All included subjects had more than 80% round-headed spermatozoa, with less than 5% morphologically normal spermatozoa with a normal 46,XY karyotype and none carried the common *DPY19L2* deletion accounting for 80% of all identified pathological alleles in globlozoospermia patients^14^. All patients gave their informed consent for the investigation of genetic analyses. Genomic DNA was extracted using standard methods, diluted to 8 ng/μl concentration, and 2 μl used for PCR amplification of individual exons of *KIAA0319L*, starting with exon 2 where the translation start codon lies. This was followed by Sanger sequencing using general methods, and mutation screening using CodonCode Aligner 6.0.2 (Codon Code Corporation). Primers used for PCR and sequencing are listed in Supplementary Table S1.

### Data availability

Most data generated during this study are included in this published article (and its Supplementary Information files). Further information may be obtained from the corresponding authors on reasonable request.

## Electronic supplementary material

Supplementary Information

## References

[1] Clermont Y (1972). Kinetics of spermatogenesis in mammals: seminiferous epithelium cycle and spermatogonial renewal. Physiol Rev..

[2] Hess, R. A. & Renato de Franca, L. Spermatogenesis and cycle of the seminiferous epithelium. *Adv Exp Med Biol.* **636**, 1–15, 10.1007/978-0-387-09597-4_1 (2008). 10.1007/978-0-387-09597-4_1 19856159

[3] Xiao X, Mruk DD, Wong CK, Cheng CY (2014). Germ cell transport across the seminiferous epithelium during spermatogenesis. Physiology (Bethesda)..

[4] Griswold MDS (2016). The Commitment to Meiosis. Physiol Rev..

[5] Abou-Haila A, Tulsiani DR (2000). Mammalian sperm acrosome: formation, contents, and function. Arch Biochem Biophys..

[6] Moreno RD, Alvarado CP (2006). The mammalian acrosome as a secretory lysosome: new and old evidence. Mol Reprod Dev..

[7] Berruti G (2016). Towards defining an ‘origin’-The case for the mammalian acrosome. Semin Cell Dev Biol..

[8] Leblond CP, Clermont Y (1952). Spermiogenesis of rat, mouse, hamster and guinea pig as revealed by the periodic acid-fuchsin sulfurous acid technique. Am J Anat..

[9] Dam AH (2007). Globozoospermia revisited. Hum Reprod Update..

[10] Coutton C, Escoffier J, Martinez G, Arnoult C, Ray PF (2015). Teratozoospermia: spotlight on the main genetic actors in the human. Hum Reprod Update..

[11] Ray PF (2017). Genetic abnormalities leading to qualitative defects of sperm morphology or function. Clin Genet..

[12] Harbuz R (2011). A recurrent deletion of DPY19L2 causes infertility in man by blocking sperm head elongation and acrosome formation. Am J Hum Genet..

[13] Koscinski I (2011). DPY19L2 deletion as a major cause of globozoospermia. Am J Hum Genet..

[14] Coutton C (2012). MLPA and sequence analysis of DPY19L2 reveals point mutations causing globozoospermia. Hum Reprod..

[15] Elinati E (2012). Globozoospermia is mainly due to DPY19L2 deletion via non-allelic homologous recombination involving two recombination hotspots. Hum Mol Genet..

[16] Zhu F, Gong F, Lin G, Lu G (2013). DPY19L2 gene mutations are a major cause of globozoospermia: identification of three novel point mutations. Mol Hum Reprod..

[17] Ghedir H (2016). Identification of a new DPY19L2 mutation and a better definition of DPY19L2 deletion breakpoints leading to globozoospermia. Mol Hum Reprod..

[18] Dam AH (2007). Homozygous mutation in SPATA16 is associated with male infertility in human globozoospermia. Am J Hum Genet..

[19] Karaca N (2014). First successful pregnancy in a globozoospermic patient having homozygous mutation in SPATA16. Fertil Steril..

[20] Elinati E (2016). A new mutation identified in SPATA16 in two globozoospermic patients. J Assist Reprod Genet..

[21] Liu G, Shi QW, Lu GX (2010). A newly discovered mutation in PICK1 in a human with globozoospermia. Asian J Androl..

[22] Yatsenko AN (2012). Association of mutations in the zona pellucida binding protein 1 (ZPBP1) gene with abnormal sperm head morphology in infertile men. Mol Hum Reprod..

[23] Yan W (2009). Male infertility caused by spermiogenic defects: lessons from gene knockouts. Mol Cell Endocrinol..

[24] de Boer P, de Vries M, Ramos L (2015). A mutation study of sperm head shape and motility in the mouse: lessons for the clinic. Andrology..

[25] Couto JM (2008). The KIAA0319-like (KIAA0319L) gene on chromosome 1p34 as a candidate for reading disabilities. J Neurogenet..

[26] Wang HZ, Qin HD, Guo W, Samuels J, Shugart YY (2013). New insights into the genetic mechanism of IQ in autism spectrum disorders. Front Genet..

[27] Eicher JD, Gruen JR (2015). Language impairment and dyslexia genes influence language skills in children with autism spectrum disorders. Autism Res..

[28] Pillay S (2016). An essential receptor for adeno-associated virus infection. Nature..

[29] Martinez-Garay I (2017). Normal radial migration and lamination are maintained in dyslexia-susceptibility candidate gene homolog Kiaa0319 knockout mice. Brain Struct Funct..

[30] Guidi LG (2017). Knockout mice for dyslexia susceptibility gene homologs *KIAA0319* and *KIAA0319L* have unaffected neuronal migration but display abnormal auditory processing. Cereb Cortex..

[31] Shima JE, McLean DJ, McCarrey JR, Griswold MD (2004). The murine testicular transcriptome: characterizing gene expression in the testis during the progression of spermatogenesis. Biol Reprod..

[32] Laiho, A., Kotaja, N., Gyenesei, A. & Sironen, A. Transcriptome profiling of the murine testis during the first wave of spermatogenesis. *PLoS One*. **8**, e61558, 10.1371/journal.pone.0061558 (2013). 10.1371/journal.pone.0061558 PMC3629203 23613874

[33] Sanz E (2013). RiboTag analysis of activelytranslated mRNAs in Sertoli and Leydig cells *in vivo*. PLoS One..

[34] Kierszenbaum AL, Tres LL (2004). The acrosome-acroplaxome-manchette complex and the shaping of the spermatid head. Arch Histol Cytol..

[35] Griswold MD (1998). The central role of Sertoli cells in spermatogenesis. Semin Cell Dev Biol..

[36] Franca LR, Hess RA, Dufour JM, Hofmann MC, Griswold MD (2016). The Sertoli cell: one hundred fifty years of beauty and plasticity. Andrology..

[37] Kierszenbaum AL, Tres LL, Rivkin E, Kang-Decker N, van Deursen JM (2004). The acroplaxome is the docking site of Golgi-derived myosin Va/Rab27a/b- containing proacrosomal vesicles in wild-type and Hrb mutant mouse spermatids. Biol Reprod..

[38] Roqueta-Rivera M, Abbott TL, Sivaguru M, Hess RA, Nakamura MT (2011). Deficiency in the omega-3 fatty acid pathway results in failure of acrosome biogenesis in mice. Biol Reprod..

[39] Hirst J (2012). Distinct and overlapping roles for AP-1 and GGAs revealed by the “knocksideways” system. Curr Biol..

[40] Kang-Decker N, Mantchev GT, Juneja SC, McNiven MA, van Deursen JM (2001). Lack of acrosome formation in Hrb-deficient mice. Science..

[41] Yao R (2002). Lack of acrosome formation in mice lacking a Golgi protein, GOPC. Proc Natl Acad Sci USA.

[42] Xiao N (2009). PICK1 deficiency causes male infertility in mice by disrupting acrosome formation. J Clin Invest..

[43] Audouard C, Christians E (2011). Hsp90beta1 knockout targeted to male germline: a mouse model for globozoospermia. Fertil Steril..

[44] Pierre V (2012). Absence of Dpy19l2, a new inner nuclear membrane protein, causes globozoospermia in mice by preventing the anchoring of the acrosome to the nucleus. Development..

[45] Wang H (2014). Atg7 is required for acrosome biogenesis during spermatogenesis in mice. Cell Res..

[46] Lin YN, Roy A, Yan W, Burns KH, Matzuk MM (2007). Loss of zona pellucida binding proteins in the acrosomal matrix disrupts acrosome biogenesis and sperm morphogenesis. Mol Cell Biol..

[47] Fujihara Y (2012). SPACA1-deficient male mice are infertile with abnormally shaped sperm heads reminiscent of globozoospermia. Development..

[48] Funaki, T. *et al*. The Arf GAP SMAP2 is necessary for organized vesicle budding from the trans-Golgi network and subsequent acrosome formation in spermiogenesis. *Mol Biol Cell.* **24**, 2633–2644, 10.1091/mbc.E13-05-0234 (2013). 10.1091/mbc.E13-05-0234 PMC3756916 23864717

[49] Doran J (2016). Mfsd14a (Hiat1) gene disruption causes globozoospermia and infertility in male mice. Reproduction..

[50] Lerer-Goldshtein T (2010). TMF/ARA160: A key regulator of sperm development. Dev Biol..

[51] Han F (2017). Globozoospermia and lack of acrosome formation in GM130-deficient mice. Cell Death Dis..

[52] Paiardi C, Pasini ME, Gioria M, Berruti G (2011). Failure of acrosome formation and globozoospermia in the wobbler mouse, a Vps54 spontaneous recessive mutant. Spermatogenesis..

[53] Gioria M, Pasini ME, Berruti G (2017). Dynamic of contribution of UBPy-sorted cargo to acrosome biogenesis: effects of its derailment in a mouse model of globozoospermia, the infertile Vps54 (L967Q) mutant. Cell Tissue Res..

[54] Suzuki-Toyota F (2004). The coiled tail of the round-headed spermatozoa appears during epididymal passage in GOPC-deficient mice. Arch Histol Cytol..

[55] Suzuki-Toyota F (2007). Factors maintaining normal sperm tail structure during epididymal maturation studied in Gopc−/− mice. Biol Reprod..

[56] Nakamura N (2010). Emerging new roles of GM130, a cis-Golgi matrix protein, in higher order cell functions. J Pharmacol Sci..

[57] Fridmann-Sirkis Y, Siniossoglou S, Pelham HR (2004). TMF is a golgin that binds Rab6 and influences Golgi morphology. BMC Cell Biol..

[58] Liewen H (2005). Characterization of the human GARP (Golgi associated retrograde protein) complex. Exp Cell Res..

[59] He J, Xia M, Tsang WH, Chow KL, Xia J (2015). ICA1L forms BAR-domain complexes with PICK1 and is crucial for acrosome formation in spermiogenesis. J Cell Sci..

[60] Lu R, Stewart L, Wilson JM (2015). Scaffolding protein GOPC regulates tight junction structure. Cell Tissue Res..

[61] Kherraf ZE (2017). SPINK2 deficiency causes infertility by inducing sperm defects in heterozygotes and azoospermia in homozygotes. EMBO Mol Med..

[62] Ibraghimov-Beskrovnaya O (2000). Strong homophilic interactions of the Ig-like domains of polycystin-1, the protein product of an autosomal dominant polycystic kidney disease gene, PKD1. Hum Mol Genet..

[63] Yan HH, Mruk DD, Lee WM, Cheng CY (2007). Ectoplasmic specialization: a friend or a foe of spermatogenesis?. Bioessays..

[64] Wong EW, Mruk DD, Cheng CY (2008). Biology and regulation of ectoplasmic specialization, an atypical adherens junction type, in the testis. Biochim Biophys Acta..

[65] Berruti G, Paiardi C (2014). The dynamic of the apical ectoplasmic specialization between spermatids and Sertoli cells: the case of the small GTPase Rap1. Biomed Res Int..

[66] Griffiths G, Warren G, Stuhlfauth I, Jockusch BM (1981). The role of clathrin-coated vesicles in acrosome formation. Eur J Cell Biol..

[67] Moreno RD, Ramalho-Santos J, Sutovsky P, Chan EK, Schatten G (2000). Vesicular traffic and golgi apparatus dynamics during mammalian spermatogenesis: implications for acrosome architecture. Biol Reprod..

[68] Ramalho-Santos J, Moreno RD, Wessel GM, Chan EK, Schatten G (2001). Membrane trafficking machinery components associated with the mammalian acrosome during spermiogenesis. Exp Cell Res..

[69] Cai H, Reinisch K, Ferro-Novick S (2007). Coats, tethers, Rabs, and SNAREs work together to mediate the intracellular destination of a transport vesicle. Dev Cell..

[70] Velayos-Baeza A, Toma C, Paracchini S, Monaco AP (2008). The dyslexia-associated gene KIAA0319 encodes highly N- and O-glycosylated plasma membrane and secreted isoforms. Hum Mol Genet..

[71] Velayos-Baeza A, Levecque C, Kobayashi K, Holloway ZG, Monaco AP (2010). The dyslexia-associated KIAA0319 protein undergoes proteolytic processing with {gamma}-secretase-independent intramembrane cleavage. J Biol Chem..

[72] Levecque C, Velayos-Baeza A, Holloway ZG, Monaco AP (2009). The dyslexia-associated protein KIAA0319 interacts with adaptor protein 2 and follows the classical clathrin-mediated endocytosis pathway. Am J Physiol Cell Physiol..

[73] Piperno G, Fuller MT (1985). Monoclonal antibodies specific for an acetylated form of alpha-tubulin recognize the antigen in cilia and flagella from a variety of organisms. J Cell Biol..

[74] De Gendt K (2011). Expression of Tubb3, a beta-tubulin isotype, is regulated by androgens in mouse and rat Sertoli cells. Biol Reprod..

[75] Hermo L, Pelletier RM, Cyr DG, Smith CE (2010). Surfing the wave, cycle, life history, and genes/proteins expressed by testicular germ cells. Part 2: changes in spermatid organelles associated with development of spermatozoa. Microsc Res Tech..

[76] Fischer, E. R., Hansen, B. T., Nair, V., Hoyt, F. H. & Dorward, D. W. Scanning electron microscopy. *Curr Protoc Microbiol*. Chapter 2, Unit2B 2, 10.1002/9780471729259.mc02b02s25 (2012). 10.1002/9780471729259.mc02b02s25 PMC3352184 22549162

[77] Graham L, Orenstein JM (2007). Processing tissue and cells for transmission electron microscopy in diagnostic pathology and research. Nat Protoc..

[78] Kotaja N (2004). Preparation, isolation and characterization of stage-specific spermatogenic cells for cellular and molecular analysis. Nat Methods..

