# Supplementary material for: AU040320 deficiency leads to disruption of acrosome biogenesis and infertility in homozygous mutant mice
Source: Sci Rep. 2018 Jul 10;8:10379. doi: 10.1038/s41598-018-28666-6 (PMC6039479; doi:10.1038/s41598-018-28666-6)
Supplement: Supplementary file 1 — Supplementary Information [file 41598_2018_28666_MOESM1_ESM.pdf]

AU040320 deficiency leads to disruption of acrosome biogenesis and infertility in homozygous mutant mice

Luiz G. Guidi[1,2], Zoe G. Holloway[1], Christophe Arnoult[3], Pierre F. Ray[3,4,], Anthony P. Monaco[1,5], Zoltán Molnár*[2], Antonio Velayos-Baeza*[1,2]

1. Wellcome Centre for Human Genetics, University of Oxford, Oxford OX3 7BN, UK
2. Department of Physiology, Anatomy, and Genetics, University of Oxford, Oxford OX1 3QX, UK
3. Genetic Epigenetic and Therapies of Infertility, Institute for Advanced Biosciences, Inserm U1209, CNRS UMR 5309, Université Grenoble Alpes, Grenoble, F-38000, France
4. UM GI-DPI, CHU Grenoble Alpes, Grenoble, F-38000, France
5. Office of the President, Ballou Hall, Tufts University, Medford, MA 02155, USA

SUPPLEMENTARY INFORMATION

Table S1. Primers used for mutation screening in *KIAA0319L* gene.

Name	Sequence	Reverse complementary to
**KLg02F1**	GTGCAACCTCTTCTCCTCAA	
**KLg03F2**	GGTCTCCATGGCAGGAACTC	
**KLg04F1**	GGCGTGAGCCACCATGCTTA	
**KLg05F1**	GAAGCCACTGAGATGTGAGA	
**KLg06F1**	AGCTGAGTCAGCAGCATTCG	
**KLg07F1**	GCTGGTCTTGAACTCAGGAA	
**KLg08F1**	CTGCCTCCTGTCAGATCAGT	
**KLg09F1**	GGTTGCCTGTGTTGGAGTTC	
**KLg10F1**	GCAGTGCCTAGCACATCATA	
**KLg11F1**	AGACTGGCCTTGGTAATCTC	
**KLg12F1**	CAGAAGGCACCGATCCATAG	
**KLg13F1**	GACCAGCAGGCAGCCTGTAT	
**KLg14F1**	TGCAGACCTCCAGACATAAG	
**KLg15F1**	GGCTGCAATGCTGTTCCGTA	
**KLg16F1**	GCACTGCCAGTTGCACAGAG	
**KLg17F1**	GATCTGGCTCTGCCATAATC	
**KLg18F1**	AAGTGAAGACAGGCTGGAAG	
**KLg19F1**	CCGTGCTGTAGCCTAATGGA	
**KLg20F1**	TCCGTGCCAAGGTGGTCATA	
**KLg21F1**	GGTGTGCTCTCATCCGTACT	
**KLg02R1**	TGCTGAGTGCTGTGCTACAT	ATGTAGCACAGCACTCAGCA
**KLg03R1**	CCGCAAGCATCAGCTTCATT	AATGAAGCTGATGCTTGCGG
**KLg04R1**	TAAGCTGGAGGCCTGCTGAG	CTCAGCAGGCCTCCAGCTTA
**KLg05R1**	CCAGGAGTGGTAGTGTATGC	GCATACACTACCACTCCTGG
**KLg06R1**	ACCATGCTGGTACAGTCTTC	GAAGACTGTACCAGCATGGT
**KLg07R1**	ATGTGTCCAGCTCAGCATTC	GAATGCTGAGCTGGACACAT
**KLg08R1**	AAGAAGGCCTGTCTGGTGTG	CACACCAGACAGGCCTTCTT
**KLg09R1**	CTGTGGCAGATCCTGATCTA	TAGATCAGGATCTGCCACAG
**KLg10R1**	ATTACAGGCGCACGCCACTA	TAGTGGCGTGCGCCTGTAAT
**KLg11R1**	AAGTCACCAGAGGCTCTATG	CATAGAGCCTCTGGTGACTT
**KLg12R1**	CCATCCTGCCTCTCAAGTGT	ACACTTGAGAGGCAGGATGG
**KLg13R2**	GTCTGGAGGTCTGCATATCT	AGATATGCAGACCTCCAGAC
**KLg14R1**	GCCTACAACTGCTAGGATCA	TGATCCTAGCAGTTGTAGGC
**KLg15R1**	TGGAACAGACGGTCTCTATG	CATAGAGACCGTCTGTTCCA
**KLg16R1**	CGCATGTCACCTGCACTAGA	TCTAGTGCAGGTGACATGCG
**KLg17R1**	GCTCACAGCCTTCTGAACAC	GTGTTCAGAAGGCTGTGAGC
**KLg18R1**	GGCTGTAACTGGCATTGGAT	ATCCAATGCCAGTTACAGCC
**KLg19R1**	GGCTACAGTGTGGTGCATTA	TAATGCACCACACTGTAGCC
**KLg20R1**	AGCGGAAGTAGCACGCGAAG	CTTCGCGTGCTACTTCCGCT
**KLg21R1**	CCTGGAGGACGTCTCTGGAT	ATCCAGAGACGTCCTCCAGG

Supplementary Figure Legends

Figure S1. Normal meiotic progression of spermatocytes is unaffected following AU040320 KO. Immunostaining of dissociated spermatocytes from wild-type and AU040320-deficient mice labelling of XY bodies with γ-H2AX (red) and paired chromatids with SCP3 (green) revealed no gross abnormalities in chromatid rearrangement during meiosis. Scale bar: 10 µm.

Figure S2. Ultrastructural analysis of epididymal sperm of AU040320-deficient mice. TEM images of epididymis from wild-type (A-D, J-N) and AU040320-deficient (E-I, O-V) mice were taken at different magnifications. Large field images (A-C, E-G) show evident differences in concentration and general morphology of spermatozoa between both type of samples. High magnification images from wild-type samples (D, J-N) show the typical dense elongated nuclei and surrounding acrosome (arrowheads), as well as mid-piece and distant sections of the sperm tail. Similar images from KO mouse samples (H, I, O-V) evidence aberrant cells with a globular or irregular nucleus lacking the acrosome, often with clearly disarranged mitochondria (H, I, O, V), although the typical mid-piece structure (asterisks) can also be detected; the tail often appears coiled around the sperm head (Q, S, U), with multiple transversal sections of the same tail (arrows) being easily detected in many cases (H, I, P, R). This morphology is similar to that reported for the *Gopc* KO mice[54-55]. n, nucleus; p, perforatorium; m, mitochondria. Scale bars: (A, B, E, F) 20 µm, (C, G) 10 µm, (D, H, I) 5 µm; (J-V) 2 µm.

Figure S3. Cytoskeletal arrangement of Sertoli cells and developing manchette appears unaltered in the absence of AU040320. Immunohistochemistry labelling the microtubular supporting structures of Sertoli cells (β-tubulin III, green) in seminiferous tubules shows no evident differences between wild-type (+/+, left panels) and AU040320-deficient (-/-, right panels) mice. (B) Labelling of the manchette (β-tubulin, red) in developing spermatids at different stages (top and bottom panels) reveals similar patterns in +/+ and -/- samples. Scale bars: (A) 50 µm; (B) 12 µm.

Figure S4. Detection of endogenous AU040320 protein by immunohistochemistry. (A-F) Double immunostaining of seminiferous tubules cross-sections to detect AU040320 in wild-type samples (A-C), using *AU040320*-KO samples as a control (D-F), with antibodies against the human homologous protein KIAA0319L raised in guinea pig (#78, green; B,E) and in rabbit (Rb-AU, red; C,F) displays a juxtanuclear overlapping signal in

germ cells (A) that disappears in control samples (D), confirming the specificity of the signal obtained in the wild-type samples. (G-J) Immunostaining with antiserum #78 (green) confirms that most signal detected in wild-type samples (G-H) disappears in the mutant cells of the seminiferous tubules (I-J), although some unspecific reactivity is still present in Leydig cells (J''). Scale bars: (D) 10 µm; (D', E, F) 5 µm; (I, J) 25 µm; (J') 12.5 µm; (J'') 6 µm.

Figure S5. Detection of endogenous AU040320 protein by western blotting. (A) Full blots for results shown in Figure 1B. Lysates (20 µg loaded per lane) from testis of wild-type (lane 1) and *AU040320* KO (lane 2) mice and from epidydimis (lane 3) and sperm (lane 4) of wild-type animals were analyzed by western blotting with specific antibody anti-KL (#78) (left blot), and with anti-actin as a control (right blot, top section). Please note that right blot was split just below the 40 kDa marker; the lower section of this blot was checked for the mitochondrial marker Tom20 (BD Transduction Laboratories, # 612278), not shown in Fig. 1B. Arrow indicates specific band for AU040320 protein. Images after Ponceau staining of these membranes are displayed on the left, further confirming a similar amount of loaded protein, and showing the Spectra Multicolor Broad Range Protein Ladder (Fermentas); apparent molecular weight (kDa) of each protein band is indicated on the sides. (B) Comparison of WB results with two specific anti-KL antibodies. Lysates (20 µg loaded per lane) from whole (as in *A*) or from decapsulated testes of wild-type (lanes 1 and 1d, respectively) and *AU040320* KO (lanes 2 and 2d, respectively) mice were analyzed by western blotting with specific anti-KL antibodies: custom guinea pig antiserum (#78) (left blot) and commercial rabbit antibody (Rb-AU) (right blot). Same specific band (*arrow*) is detected by both antibodies in wild-type lysates, which is absent in KO lysates. A number of other bands, different for each antibody, are detected in both wild-type and KO lysates suggesting these correspond to unspecific signal. No difference is detected between whole and decapsulated lysates. Ponceau staining panels confirm similar amount of loaded protein in all lanes. Protein ladder as in *A*.

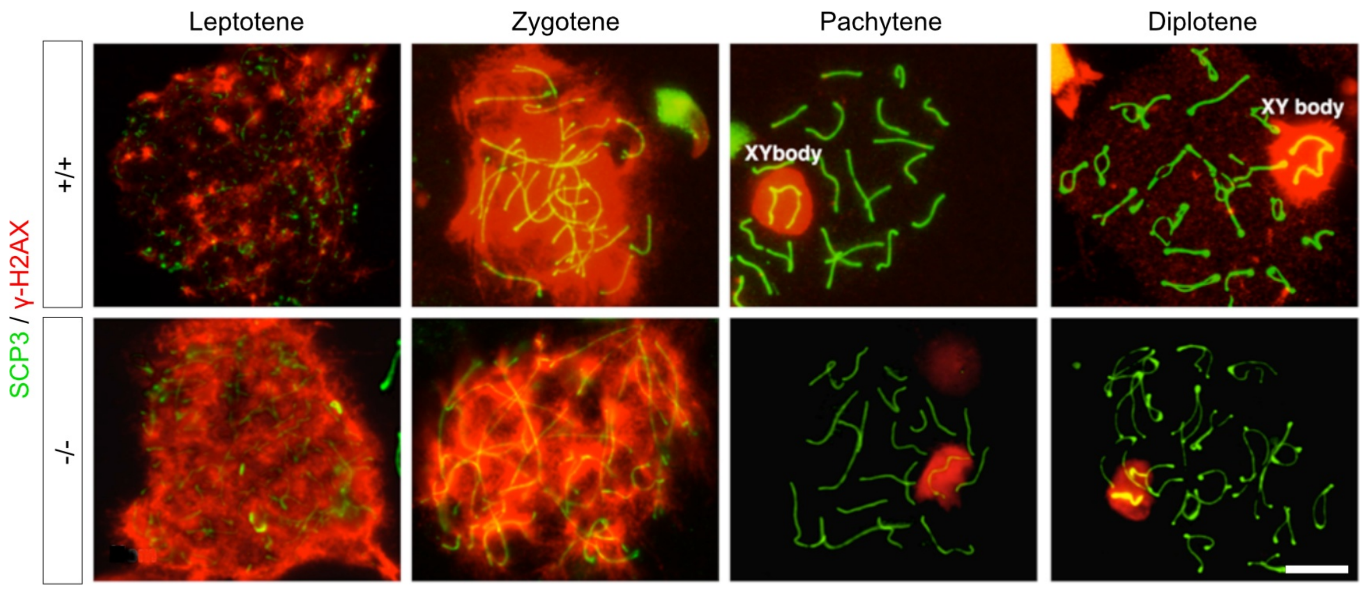

Leptotene
Zygotene
Pachytene
Diplotene
SCP3 / γ-H2AX
+/+
-/-
XYbody
XY body

Figure S1.

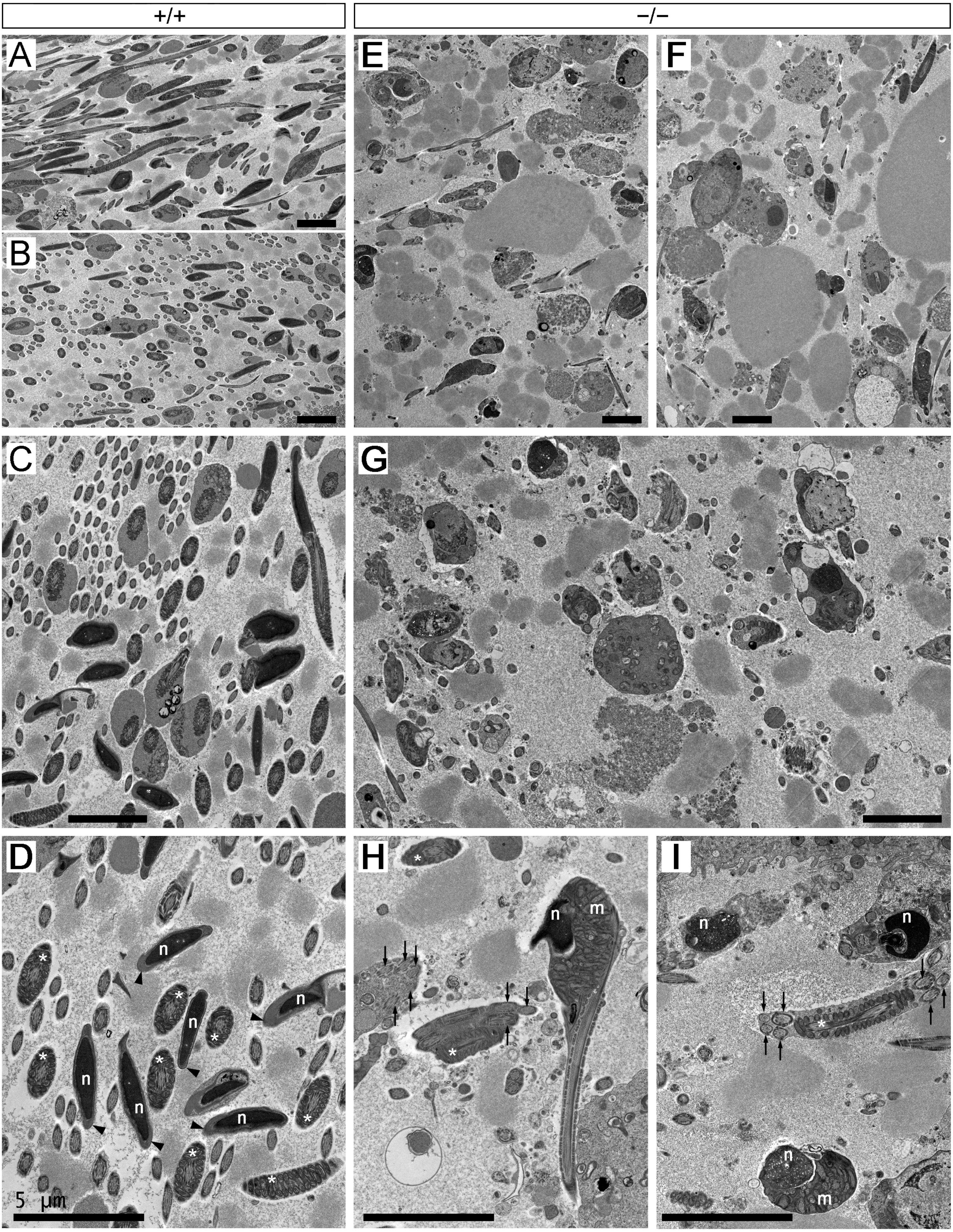

Figure S2

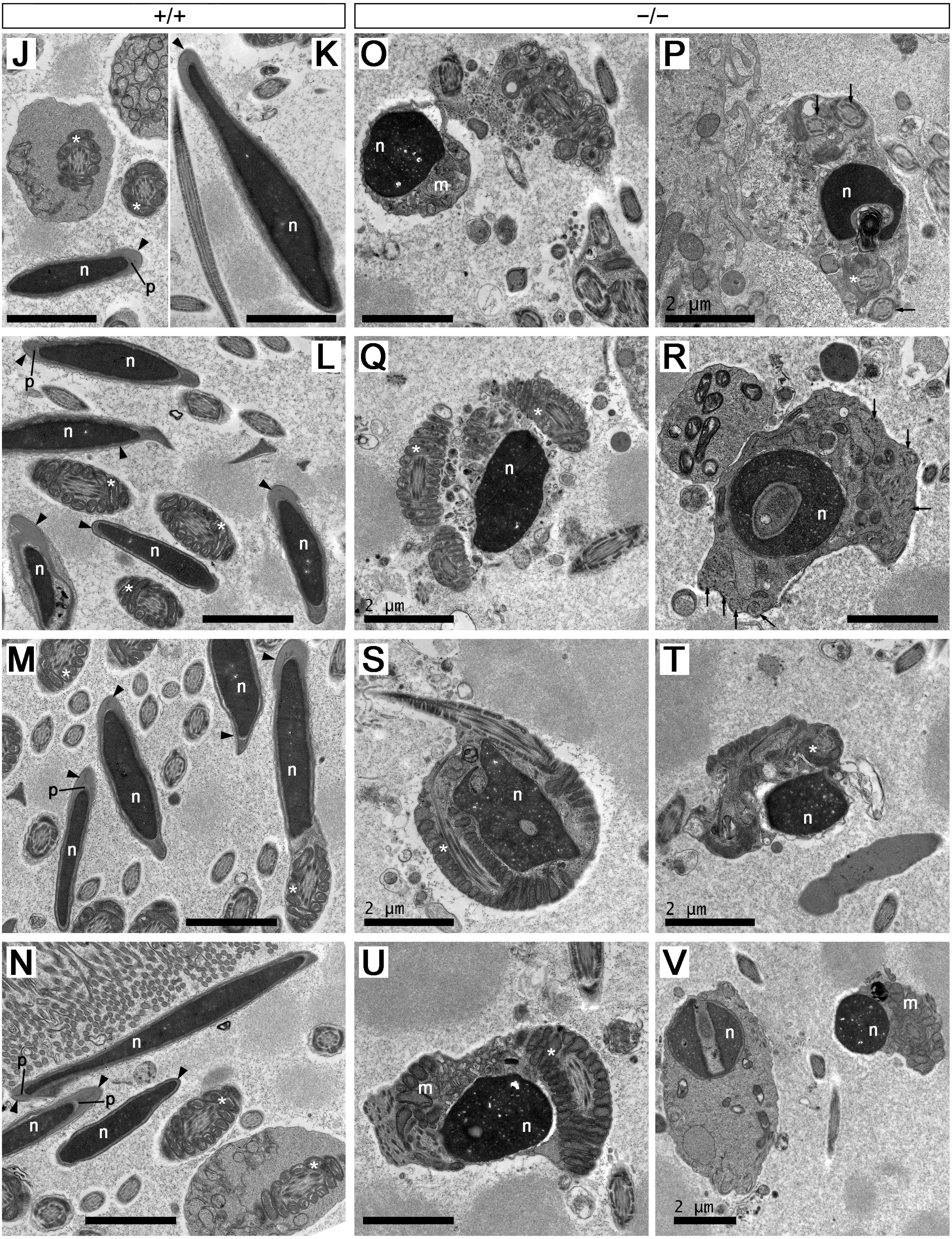

Figure S2 (cont.)

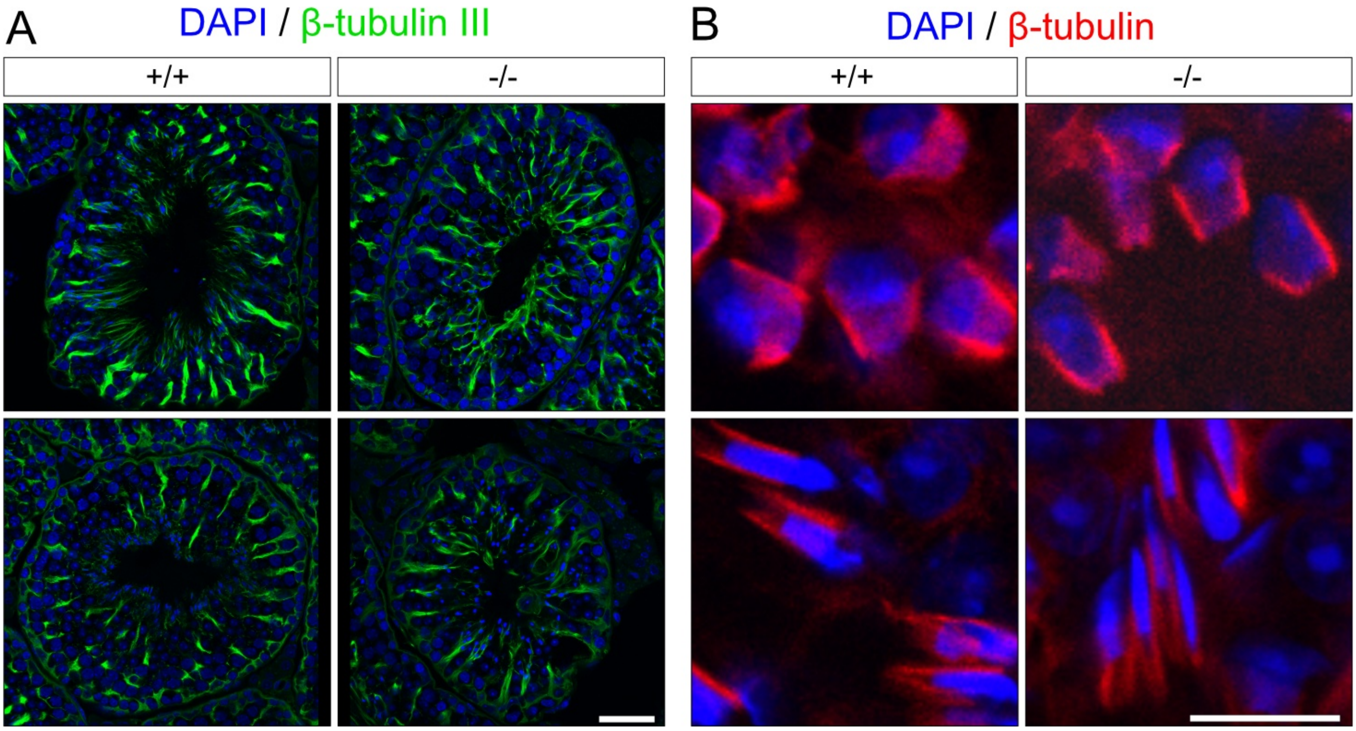

Figure S3.

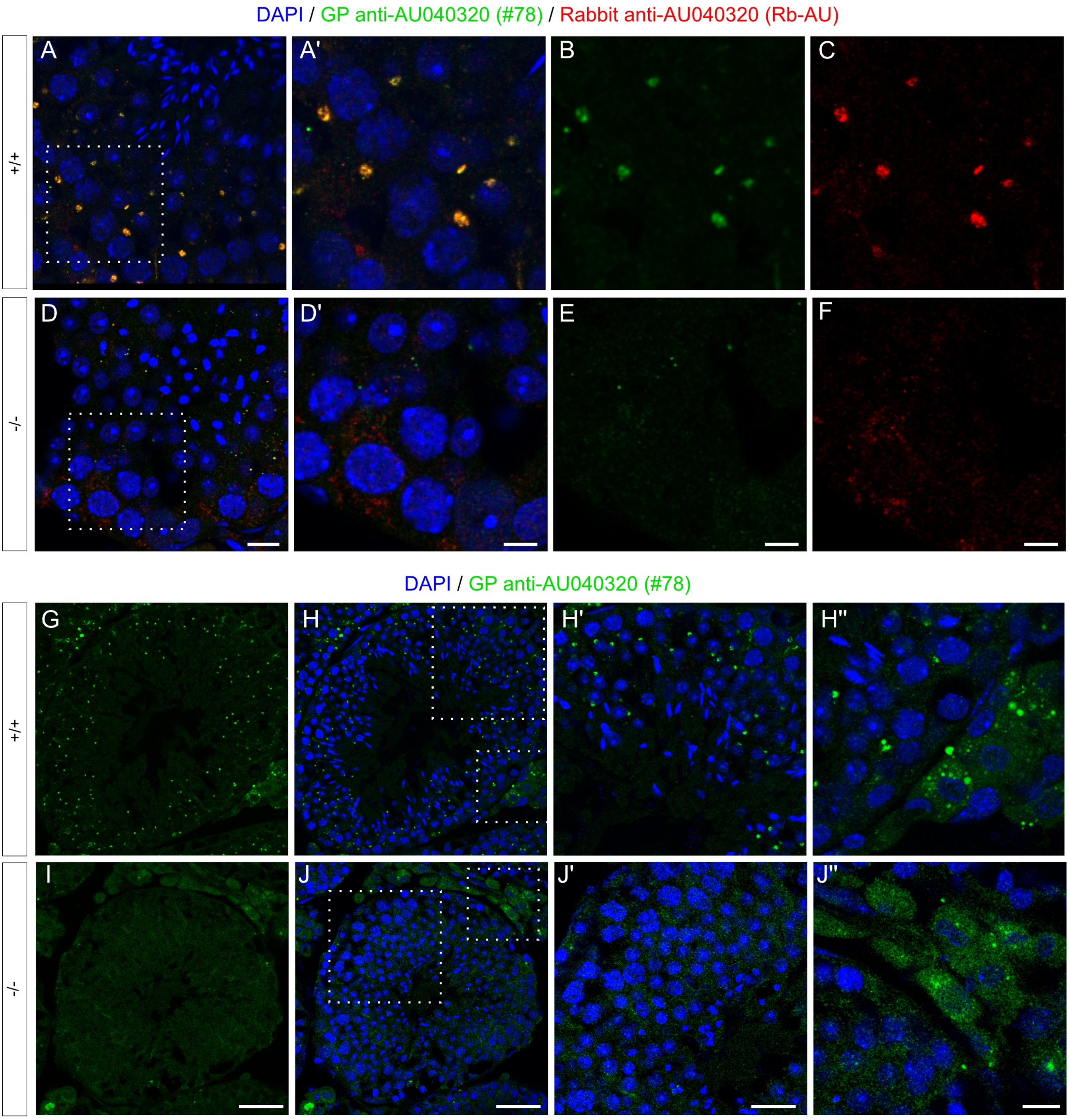
DAPI / GP anti-AU040320 (#78) / Rabbit anti-AU040320 (Rb-AU)
+/+
A
A'
B
C
-/-
D
D'
E
F
DAPI / GP anti-AU040320 (#78)
+/+
G
H
H'
H''
-/-
I
J
J'
J''

Figure S4.

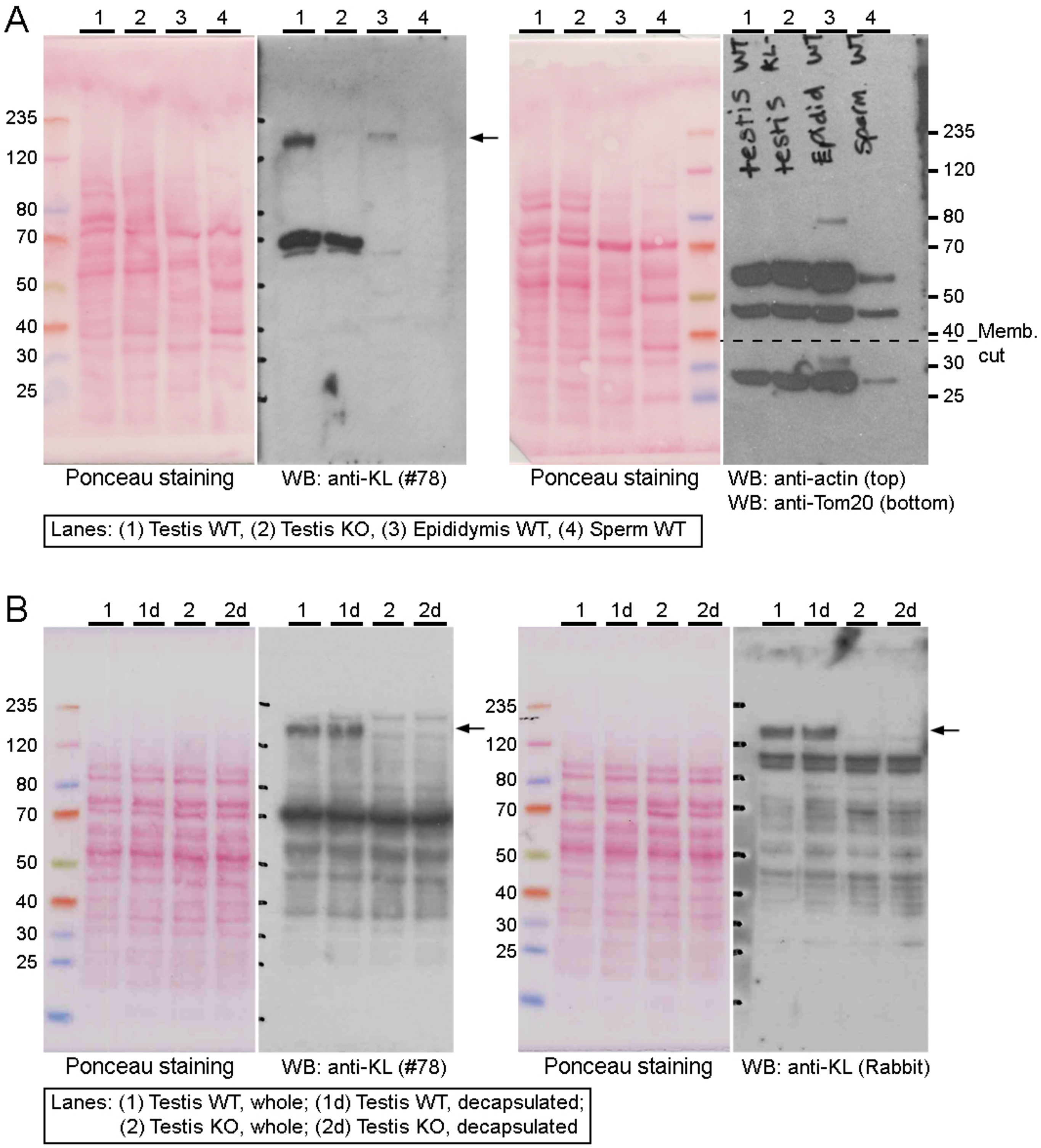
A
1 2 3 4
235
120
80
70
50
40
30
25
Ponceau staining
WB: anti-KL (#78)
Ponceau staining
WB: anti-actin (top)
WB: anti-Tom20 (bottom)
Memb. cut
Lanes: (1) Testis WT, (2) Testis KO, (3) Epididymis WT, (4) Sperm WT
B
1 1d 2 2d
Ponceau staining
WB: anti-KL (#78)
Ponceau staining
WB: anti-KL (Rabbit)
Lanes: (1) Testis WT, whole; (1d) Testis WT, decapsulated;
(2) Testis KO, whole; (2d) Testis KO, decapsulated

Figure S5
